# Chromosomal Translocations in NK-Cell Lymphomas Originate from Inter-Chromosomal Contacts of Active rDNA Clusters Possessing Hot Spots of DSBs

**DOI:** 10.3390/cancers13153889

**Published:** 2021-08-02

**Authors:** Nickolai A. Tchurikov, Leonid A. Uroshlev, Elena S. Klushevskaya, Ildar R. Alembekov, Maria A. Lagarkova, Galina I. Kravatskaya, Vsevolod Y. Makeev, Yuri V. Kravatsky

**Affiliations:** 1 Engelhardt Institute of Molecular Biology Russian Academy of Sciences, 119334 Moscow, Russia; giedre@inbox.ru (E.S.K.); alembeki@gmail.com (I.R.A.); galina.kravatskaya@gmail.com (G.I.K.); vsevolod.makeev@gmail.com (V.Y.M.); jiri@eimb.ru (Y.V.K.); 2 Vavilov Institute of General Genetics Russian Academy of Sciences, 119991 Moscow, Russia; leoniduroshlev@gmail.com; 3 Federal Research and Clinical Center of Physical-Chemical Medicine, Federal Medical Biological Agency, 119435 Moscow, Russia; lagar@rcpcm.org; 4 Center for Precision Genome Editing and Genetic Technologies for Biomedicine, Federal Research and Clinical Center of Physical-Chemical Medicine, Federal Medical Biological Agency, 119435 Moscow, Russia; 5 Moscow Institute of Physics and Technology, State University, 141700 Dolgoprudny, Russia

**Keywords:** NK-cell lymphomas, T cells, rDNA genes, inter-chromosomal contacts, 4C, translocations

## Abstract

**Simple Summary:**

There are nine DSB hot spots located in the non-transcribed spacer of human rDNA units. Circular chromosome conformation capture data indicate that the rDNA clusters often shape contact with a specific set of chromosomal regions containing genes controlling differentiation and cancer, and often possessing the DSB hot spots. The data suggest a mechanism for rDNA-mediated translocation, and some of them could lead to tumorigenesis. Here, we searched for translocations in which rDNA clusters are involved. WGS data of normal T cells and NK-cell lymphomas from the same individuals were used. We revealed numerous translocations in which rDNA units are involved. The sites of these translocations in normal T cells and in the lymphomas were mostly different, but occurred at about the same frequency in both cell types. We conclude that oncogenic translocations lead to dysregulation of a specific set of genes controlling development.

**Abstract:**

Endogenous hot spots of DNA double-strand breaks (DSBs) are tightly linked with transcription patterns and cancer. There are nine hot spots of DSBs (denoted Pleiades) in human rDNA units that are located exclusively inside the intergenic spacer (IGS). Profiles of Pleiades coincide with the profiles of γ-H2AX, suggesting a high level of in vivo breakage inside rDNA genes. The data were confirmed by microscopic observation of the largest γ-H2AX foci inside nucleoli in interphase chromosomes. Circular chromosome conformation capture (4C) data indicate that the rDNA units often make contact with a specific set of chromosomal regions containing genes that are involved in differentiation and cancer. Interestingly, these regions also often possess hot spots of DSBs that provide the potential for Robertsonian and oncogenic translocations. In this study, we searched for translocations in which rDNA clusters are involved. The whole genome sequence (WGS) data of normal T cells and NK-cell lymphomas from the same individuals revealed numerous translocations in which Pleiades were involved. The sites of these translocations in normal T cells and in the lymphomas were mostly different, although there were also some common sites. The genes at translocations in normal cells and in lymphomas are associated with predominantly non-overlapping lists of genes that are depleted with silenced genes. Our data indicate that rDNA-mediated translocations occur at about the same frequency in the normal T cells and NK-lymphoma cells but differ at particular sites that correspond to open chromatin. We conclude that oncogenic translocations lead to dysregulation of a specific set of genes controlling development. In normal T cells and in NK cells, there are hot spots of translocations at sites possessing strong H3K27ac marks. The data indicate that Pleiades are involved in rDNA-mediated translocation.

## 1. Introduction

Chromosomal translocations are a physiologic mechanism of DNA recombination in germline cells and in lymphocyte development. Although these mechanisms have been studied for several decades, they are still not fully understood. Chromosomal translocations are common in cancers and more frequently occur between neighboring chromosomal regions [1,2]. As a result, some translocations lead to the formation of oncogenic fusion proteins or to the activation of oncogenes by the altered regulatory regions [3,4]. Ribosomal DNA (rDNA) genes are the most fragile regions in the human genome and possess nine hot spots of DSBs, each of which is about 50–100 bp in length and are denoted “Pleiades” [5,6]. Additionally, it was demonstrated by the 4C (circular chromosome conformation capture) approach that rDNA clusters shape contacts with different chromosomal regions and are involved in the epigenetic regulation of genes that are highly associated with differentiation and cancer [7]. Interestingly, rDNA-contacting sites often possess hot spots of DSBs. rDNA clusters also make frequent contacts with pericentromeric regions and regions possessing stretches of 5–50 kb that contain H3K27ac marks [5]. Therefore, Pleiades may be responsible for Robertsonian translocations, in which rDNA-containing chromosomes are necessarily involved via their broken pericentromeric regions [5]. These data indicate that rDNA genes are a candidate for mediating chromosomal translocations in normal and cancer cells. Here, we investigated whether rDNA genes are involved in chromosomal translocations by searching for rDNA-mediated translocations in normal T cells and in natural killer (NK)-cell lymphomas from the same individuals.

Natural killer cells play an important role in the innate immune system that controls several types of tumors and infections [8]. NK cells, along with B and T lymphocytes, belong to a group of innate lymphoid cells that differentiate from a common lymphoid progenitor. NK cells are closely related to T cells and can lyse target cells without prior sensitization.

Our data indicate that active rDNA clusters are involved in translocations in both types of cells, particularly in regions with actively transcribed genes, but the spectra of the target genes in these cell types differ. The same rDNA-mediated hot spots of translocations are found in normal and cancer cells corresponding to the hot spots of inter-chromosomal contacts of nucleoli within specific genomic regions.

## 2. Materials and Methods

### 2.1. Determination of rDNA-Mediated Translocation Sites in Paired-End WGS Reads

We used the following procedure to find translocations. The data from the European Genome Archive [9] were analyzed. The NK-cell lymphoma dataset (EGAD00001003271) was selected (102 matched-sample pairs, read length 100 bp, average amplicon length 300 bp). Each pair of matched samples contained samples of cancer cells (NK-cell lymphoma) and control cells (T cells) from the same donor. Paired-ended sequencing was conducted for all samples using the Illumina Genome Analyzer IIx and Illumina HiSeq 2000 resulting in a pair of FASTQ files for each sample, one file (L1) contained left terminal reads for each amplicon, and the other file (L2) contained the right terminal reads. Then, we searched for leading or anchor sequences corresponding to rDNA stretches (Table 1) located before a hot spot of DSBs in the samples. We required an exact match between the read and the leading sequence. Leading sequences were analyzed in both the left and right fragment termini (reads indexed as R1 and R2, respectively). For each leader sequence, all reads containing this sequence were stored in a separate FASTQ file. We applied the grep utility to search for leading sequences.

Table 1 contains the average number of reads for each leading sequence. The average number of reads is given for the first reads from each paired-end read pair. The Bwa [10] 0.7.17-r1188 mem algorithm was used to map sample reads for the U13369.1 sequence. For each amplicon, a SAM file was obtaining containing alignments for both amplicon termini.

For each SAM file, we used Samtools [11] 1.6 to select non-matched reads, i.e., the reads where one amplicon’s end was found in U13369.1 while the other was not. These non-matched reads were used to locate translocations using an ad hoc in-house Ruby script. Each of the non-paired amplicon ends was aligned with human genome build hg19/GRCh37.p13 to find exact matches (without deletions or substitutions). Each alignment of a non-paired amplicon’s end is presented in Table 1.

### 2.2. Genome-Wide rDNA-Mediated Translocation Mapping

The rDNA-mediated translocation-associated amplicons (Table 1) were mapped to the genome build hg19/GRCh37.p13 genome-wide by the following procedure. For each anchor, the amplicon’s parts that do not contain the anchor sequence (so it has to map to the genome other than the rDNA) were selected. These selected sequences were mapped to the genome by bowtie2 [12] 2.3.4.1 with preset–local-sensitive. The unaligned reads were removed, and the alignment file was sorted and converted to the BAM file by Samtools. The BAM file was converted to the table containing the chromosome, begin of mapping region, end of mapping region, length of mapping region, coverage of mapping region, number of reads that mapped to the region, and sequence of the region by in-house ad hoc Perl and bash scripts. The mapping table was converted to GFF format by an in-house Perl script for further epigenetic plotting.

The mapping regions were assigned to genes in the following way. Ensembl genome annotation hg19/GRCh37p.13 v.87 was used to obtain the list of genes. The gene names, IDs, and chromosome coordinates were extracted from the annotation file by the R script with the help of refGenome and dplyr libraries. The intersections between the rDNA-mediated translocation mapping file and the gene list were found by intersectBed [13] tool.

### 2.3. 4C-rDNA Experiments

DNA samples for the 4C experiments were isolated according to procedures described previously [5,7]. HEK293T, K562, and hESM01 cells were fixed in 1.5% formaldehyde, and nuclei were isolated, followed by digestion with *Eco*RI enzyme and ligation of extensively diluted DNA to favor intramolecular ligations. To shorten the ligation products, digestion with *Fae*I was performed followed by ligation of diluted DNA samples to favor circularization. The details are described in Supporting Information. The final 4C DNA samples were used for the preparation of 4C-rDNA libraries that were subjected to deep sequencing using HiSeq1500 (Illumina) using up to 250-nt long reads. The 4C-rDNA raw data corresponding to two biological replicates for each cell type were deposited under accession numbers GSE121413, GSE175909, and GSE175910.

### 2.4. RNA-Seq Analysis

We performed expression analysis for T and NK cells uniformly. T-cell RNA-Seq data (three replicates ENCSR306IAW, ENCSR336VTK, and ENCSR631FXT) and NK-cell RNA-Seq data (two replicates SRR10960490, SRR10960492) were used. The Trimmomatic [14] tool was applied to remove low-quality reads with the following options: LEADING:18 TRAILING:18 SLIDINGWINDOW:4:22 MINLEN:20. RSEM software [15] was applied to obtain accurate transcript quantification for the RNA-Seq data with the hg19/GRCh37.p13 genome and Ensembl annotation v.87 builds, and the results were averaged between replicates for each cell line. Gene expression values (in TPM) were assigned to the previously obtained rDNA-mediated translocations genes list and were used to create violin plots and heatmaps. The heatmap were created by R scripts with the help of ggplot2 and ComplexHeatMap [16] libraries.

### 2.5. 4C-rDNA Genome-Wide Mapping

4C-rDNA genome-wide mappings were performed for human cell line datasets HEK293T (GSM3434713 and GSM3434714), K562 (GSM5351031 and GSM5351032), and hESM01 (GSM5351029 and GSM5351030) uniformly. Adapters were removed according to the appropriate GEO descriptions. The filtered datasets were aligned to the hg19/GRCh37p.13 human genome by the bwa mem algorithm. Samtools was used to remove unaligned reads, sort, and convert alignment files to the BAM format. Then, BAM files were converted to the bedGraph profiles by the genomeCoverageBed [13] tool. The low complexity and/or repeat regions were removed from the profiles by subtractBed [13] tool. The regions were removed from the profiles only if they were mapped to DFAM [17] database entries completely. The final profiles represent the mean intersections between replicates for each cell line and were created by intersectBed tool.

### 2.6. Genome-Wide Profiles

The following genome-wide fold change compared to the control for hg19 profiles were downloaded from the Encode [18] project: for the T cells, H3K27me3 (ENCFF646OPC), H3K27ac (ENCFF491BKU), H3K4me3 (ENCFF692YGS), H3K4me1 (ENCFF376PDM), H3K9me3 (ENCFF331ZNM), and DNase I HSS (ENCFF334YII, ENCFF472QRS); for the NK cells, H3K27me3 (ENCFF269TUJ), H3K27ac (ENCFF238EMY), H3K4me3 (ENCFF428SHV), H3K4me1 (ENCFF204KCP), H3K9me3 (ENCFF292VWV), and DNase I HSS (ENCFF448RXA and ENCFF544OID).

All the epigenetic charts were created by the SeqPlots [19] R package interactively.

### 2.7. Epigenome Statistics

Epigenome chromatin state statistics were calculated for the core 15-state model (five marks) for the data downloaded from NIH Roadmap Epigenomics [20] for the T cells (E034 epigenome: primary T cells from peripheral blood) and NK cells (E046 epigenome: primary NK cells from peripheral blood). Intersections between rDNA-mediated translocation mappings and chromatin state coordinates were found by the intersectBed tool and statistics were calculated by the in-house Perl script.

### 2.8. Statistics

We applied the non-parametric Mann–Whitney *U*-test to verify whether rDNA-mediated translocation gene-subset expression values (expression of genes that intersected with rDNA-mediated translocations by genome-wide mapping) and the full expression dataset values originate from the same distribution. We obtained *p* = 0.0022 for the paired T-cell full expression set and translocation-associated genes subset, and *p* = 0.0013 for the paired NK-cell full expression set and translocation-associated genes subset. We obtained *p* = 0.033 for the Mann–Whitney *U*-test for the expression values of T-cell and NK-cell subsets, which means that we can reject the null hypothesis and conclude that translocation-associated gene expression value subsets are significantly different between T and NK cells.

We tested the statistical significance of the difference between epigenome states with the independent samples t-test. All cases that significantly differ are marked by asterisks.

## 3. Results

### 3.1. rDNA-Mediated Translocations Are Present in Both T Cells and in NK-Cell Lymphomas

**Figure 1:**
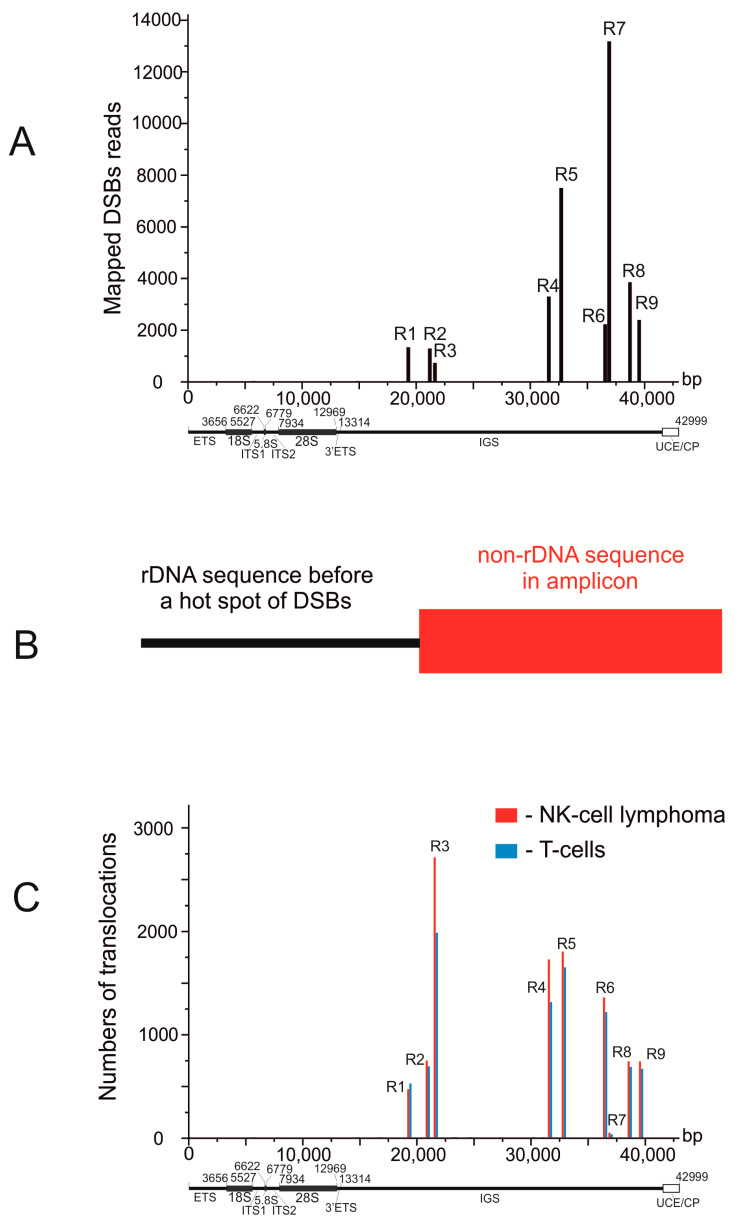
Hot spots of DSBs coincide with translocation sites in rDNA units. (**A**) mapping of DBSs along rDNA genes in HEK293T cells [21] R1–R9 are the hot spots of DSBs or Pleaides. UCE/CP: upstream promoter element and core promoter. (**B**) the scheme illustrates the search for rDNA-mediated translocations in WGS amplicons after paired-end sequencing. Amplicons possessing rDNA sequences located before Pleaides at one end and non-rDNA sequences at the other end were selected for further analysis. (**C**) mapping of the detected translocations along rDNA units in T cells and in NK-cell lymphomas. The 102 samples correspond to normal T cells and NK-cell lymphomas from the same individual.

To search for rDNA-mediated translocations, we used the dataset with accession EGAD00001003271, which contains the whole genome sequence (WGS) of T cells and NK-cell lymphoma, from the European Genome-phenome Archive. The dataset contains 102 paired samples from tumor (NK cells) and normal (T cells) cells from the same individual. The details of the search are described in the Section 2. Figure 1A shows that Pleaides (R1–R9) are located exclusively in the intergenic spacer (IGS) of rDNA units [5]. WGS amplicons that were initially used for paired-end sequencing were selected as they contain the rDNA anchor sequences. The anchors were selected using the alignments of DSB reads along the rDNA sequence (for details see the Supplementary Information in [5]). The anchors correspond to rDNA stretches located about 50 bp before the Pleaides on one side of each selected paired-ends read (Table 1, Figure 1B). Then, these amplicons were analyzed from other sides, and if non-rDNA stretches were detected there, the corresponding amplicons were considered as representing the translocation events. Amplicons possessing rDNA sequences at both ends correspond to the rDNA units.

We detected that the Pleiades coincide with translocation sites in rDNA units, which indicates that the hot spots of DSBs are involved in translocation events. The numbers of detected translocations at different hot spots of DSBs in rDNA units significantly vary (Figure 1C, Table 1).

The most frequent translocations were formed at R3. The frequencies likely depend not only on the frequencies of DSBs in rDNA but also on the DSB frequencies at a target site. The 3D structure of the rDNA units is also important for establishing the contacts with different genomic regions [5]. The translocation frequencies may also depend on the dynamics of the contacts and the distances between broken chromosomes, as well as the accessibility of non-rDNA sequences at particular sites, which is tightly connected with the expression patterns of genes in a region. We observed contrasting frequencies of DSBs and translocations inside rDNA units. The most frequent breakage in rDNA was detected in R7 and the lowest in R3. For the formation of translocations, it is likely that the activity and fidelity of DNA reparation and recombination systems in different chromosomal regions are also important. Moreover, translocation events are followed by the selection of this particular cell and its daughter cells. During this selection, an initial cancer stem cell may appear giving rise to a population of cancer cells. Table 1 shows that in the 102 pairs of samples analyzed, we detected 91 and 101 rDNA-mediated translocations per genome in T cells and NK-cell lymphomas, respectively. The frequencies of translocation in the normal and NK cells are similar, which indicates that NK-cell lymphoma tumorigenesis is not associated with the translocation frequency. In this study we used normal T cells from individuals that had lymphomas as a control. It cannot be excluded that the frequency of rDNA-mediated translocations in T cells from individuals without lymphoma is lower because the DNA repair mechanisms are more consistent in healthy organisms.

### 3.2. In T Cells and NK-Cell Lymphomas, Translocations Involve Different Sets of Genes Controlling Development

**Figure 2:**
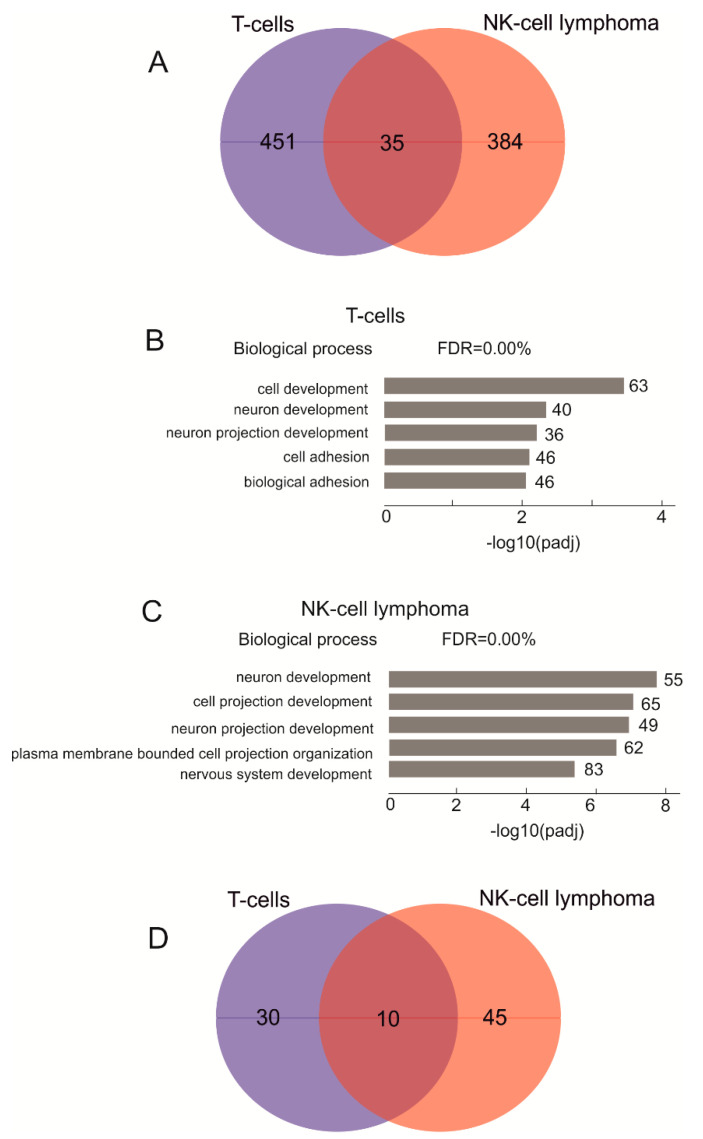
Analysis of genes located at translocation sites in T cells and NK-cell lymphomas. (**A**) a Venn diagram showing the intersections between genes at translocation sites in T cells and NK-cell lymphomas. Table S2 shows the list of overlapping genes. (**B**) the top five Gene Ontology biological process associations of genes shown in (**A**) that correspond to the affected genes in T cells. The values to the right of the bars show the number of genes associated with a process. Table S3 shows the results of the corresponding GO search. (**C**) the top five Gene Ontology biological process associations of genes shown in (**A**) that correspond to the affected genes in NK-cell lymphomas. The values to the right of the bars show the number of genes associated with a process. Table S4 shows the results of the corresponding GO search. (**D**) a Venn diagram showing the intersections between genes corresponding to GO item GO:0048666 (neuron development) at translocation sites in T cells and NK-cell lymphomas.

As we detected no connection between the translocation frequencies in normal and cancer cells, next, we searched for putative differences in genes that reside at the translocations in T cells and NK-cell lymphomas. We assumed that sites of the translocations in normal T cells and NK-cell lymphomas could differ, depending on the distinct patterns of rDNA contacts and chromatin structures at the target sites in each cell type. These differences in translocation sites may affect different sets of genes in these cells and thus give rise to either normal or cancer phenotypes. For this search, we mapped all detected translocations in the human genome using the non-rDNA halves of the amplicons (see Methods) to obtain lists of the genes located close to the translocation sites (±5 kb) in both cell types (Table S1). Previously, we observed that in three human cell lines of different origin (HEK293T, K562, and hESM01), about 50% of rDNA-contacting genes are shared between cell types [21]. Surprisingly, in this study, the translocation target genes in T cells and NK-cell lymphomas are very different (Figure 2A).

At translocations in lymphomas, we found 514 genes in 102 samples, while in T cells, in total, we detected 431 genes at these sites. Only 35 genes were common between the two cell types, while >90% of the genes at translocations were different. Although HEK293T, K562, and hESM01 cultured cells of different origin have a cancer phenotype and are capable of an unlimited number of cell division cycles, they still share about 50% of their rDNA-contacting genes. Among the genes located in the regions of translocations in NK-cell lymphomas is *FHIT* (Figure S1A). This tumor suppresser gene is very large and corresponds to the common fragile site FRA3B. There are frequent translocations at the same site (coordinate 59.67 Mb) in T cells, which is in chr3 and downstream from the *FHIT* gene. In NK-cell lymphomas, we detected two sites of more frequent translocations (up to 40 in the same sites) further downstream of the gene. Another large tumor suppressor gene that underwent translocation in NK-cell lymphomas is *WWOX* (four translocations), which corresponds to another fragile site (FRA16D; Table S1). The WWOX protein is involved in apoptosis and the mutation of *WWOX* is associated with many types of cancer. Common fragile sites are found in leukocytes [22]. We observed 38 translocations in the same position inside *CTCF* in NK-cell lymphomas (Table S1, Figure S1B). The *CTCF* gene is an epigenetic modulator of cancer [23]. No translocations affecting *WWOX* and *CTCF* in T cells were detected (Figure S1B). The data suggest that the architecture of chromosomes in lymphoma cells was changed compared with that in T cells. Inside the *KDM2A* gene, 112 translocations were detected in lymphoma cells but only 35 in T cells. (Table S1). The gene specifies lysine demethylase 2A, specific to H3K36, and is important for chromatin organization.

In order to understand the nature of the genes involved in rDNA-mediated translocations in NK-cell lymphomas and T cells, we used a Gene Ontology (GO) search. The target genes in T cells were associated with a number of biological process items relating to cell development and neuron differentiation (Figure 2B, Table S3). In NK-cell lymphomas, the genes associated with the translocations are highly associated (padj up to 10^−8^) with cell projection (Figure 2C, Table S4). Among the top five GO items in NK-cell lymphomas, we found the same groups of neuron development and neuron projection development genes that were previously detected in the normal T cells (Figure 2B). Nevertheless, even within these particular groups, there is only a small overlap between the translocation-associated genes (Figure 2D). These results confirm our conclusion that cancer cells have altered chromosomal structures that are formed by rDNA units and that chromosomal structures are tightly associated with translocations.

### 3.3. There Are Hot Spots of rDNA-Mediated Translocations in T Cells and NK-Cell Lymphomas

**Figure 3:**
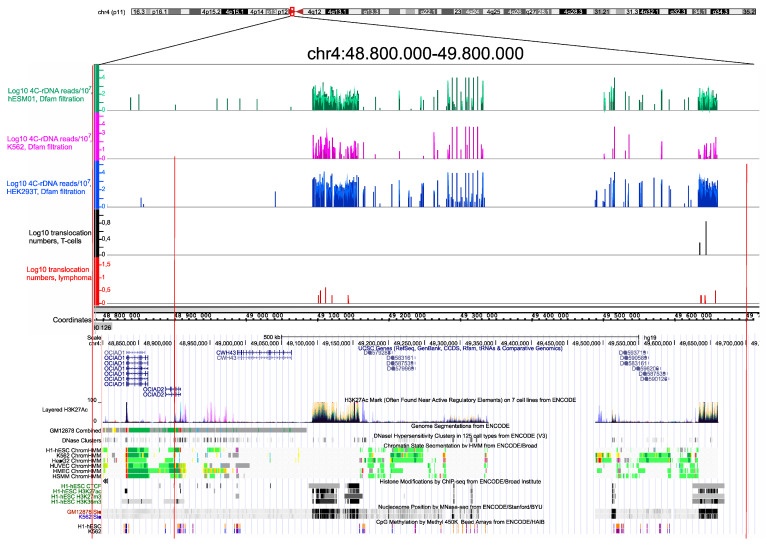
Translocation sites in both normal T cells and in NK-cell lymphomas coincide with and correspond to the hot spots of rDNA contacts in three human cell lines of different origin. The distribution of rDNA-contacting sites in K562 cells, HEK293T, and in hESM01 cells [24] in the pericentromeric region of chr4 are shown as in IGB Browser (hg19). The translocation sites detected in 102 samples, each containing amplicons from T cells and NK-cell lymphomas from the same individual, are shown. The distribution of UCSC genes, layered H3K27ac marks, genome segmentation from ENCODE, histone modifications, nucleosome position, and CpG methylation inside of a 1-Mb region of chr4 are shown as in the UCSC Browser (hg19).

Previously, it was shown that there are multiple rDNA-contacting sites that are decorated by the prominent H3K27ac marks within chromosomal regions up to 100 kb in length that probably correspond to super-enhancers [5,7]. We next determined whether these regions possess hot spots of rDNA-mediated translocations. Figure 3 shows that in both normal T cells and NK-cell lymphomas, there are translocations sites within these regions.

The translocations sites exactly correspond to the regions with very frequent rDNA contacts in three human cell lines (K562, HEK293T, and hESM01. These regions are also characterized by CTCF-binding sites and nucleosome positioning. Similarly, both the hot spot of rDNA contacts and rDNA-mediated translocation in normal and cancer cells were observed in pericentromeric regions in different human chromosomes. The nature of such chromosomal regions and the possible role of rDNA contacts at these sites are not yet known. Nevertheless, these data strongly support the proposal that frequent rDNA contacts lead to a high potential for chromosomal translocations in pericentromeric regions. The data confirm the previous data suggesting that Robertsonian translocations are the result of rDNA-mediated translocations in which rDNA-containing chromosomes are involved [5]. The fact that these abundant contacts occur in different human cell lines suggests that these chromosomal regions are involved in the formation of conserved 3D structures. The corresponding hot spots of translocations occur in both normal and cancer cells and are not associated with tumorigenesis and thus reflect the stable inter-chromosomal structures of nucleoli.

It is not clear how distant translocations could affect the neighboring genes. We detected two translocations 165–and 470-kb downstream from the 3′ end of the *FHIT* gene in NK-cell lymphomas (Figure S1). These translocations reside in the same TAD of about 900 kb in length (Figure S2). It cannot be excluded that the damage done to this domain by these translocations could affect the gene.

Interestingly, about 10% of translocation sites in both T cells and NK-cell lymphomas correspond to long non-coding RNAs (lincRNAs) (Table S1). The function of these genes is not yet known. One possible explanation is that lincRNAs, which shape chromatin structures and regulate transcription, may also be involved in the mechanisms of nucleoli inter-chromosomal contacts.

One example, *LINC00486*, which is a gene of about 100 kb in chr2, possesses 219 translocations in NK-cell lymphomas (Figure S3). The same region is also affected in the normal T cells, but here we detected only 26 translocations. The site of numerous translocations inside *LINC00486* exactly corresponds to the prominent H3K27ac mark in seven cell lines from ENCODE, and to the DNase I hypersensitive site (Figure S3). Recently, it was shown that *LINC00486* inhibits proliferation and promotes apoptosis of breast cancer tissues through the targeted regulation of miR-182-5p expression [25]. In non-small cell lung cancer cells, the *LINC00486* gene is a hot spot of breakpoints that demonstrate an extremely high rearrangement rate and might correspond to a new fragile site [26].

### 3.4. Translocation Sites in T Cells and NK-Cell Lymphomas Have Fewer Silenced Genes

**Figure 4:**
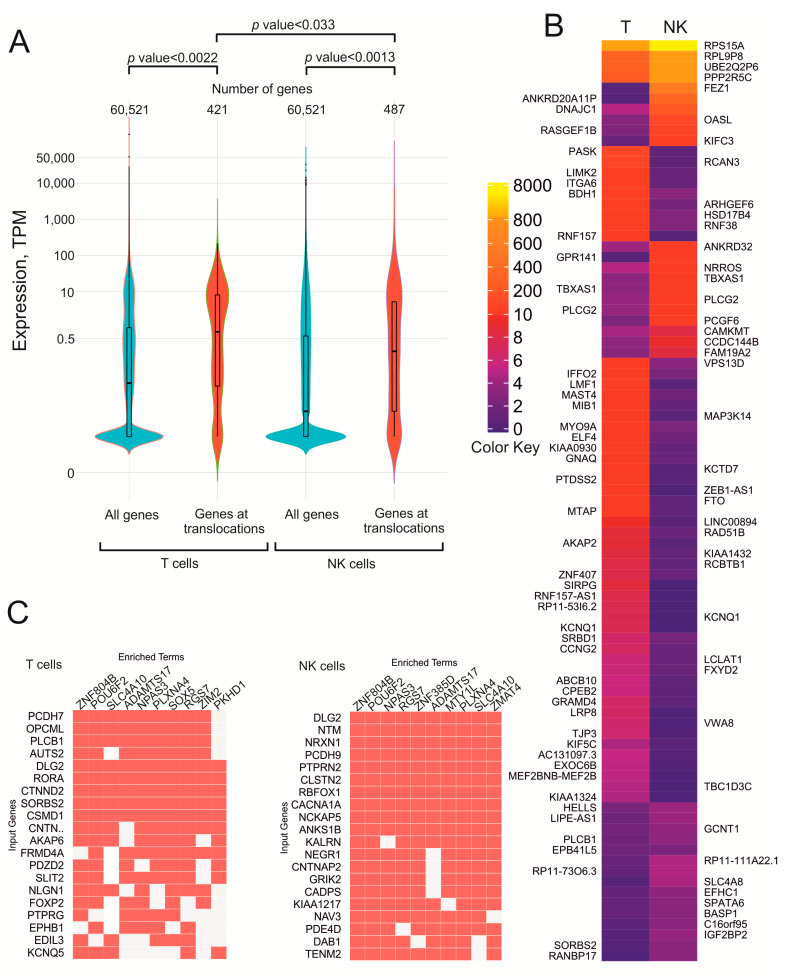
Expression of genes at translocation sites in normal T cells and in NK cells. (**A**) Violin plots showing the distribution of all genes along their expression levels (blue) and along genes at translocation sites in both cell types (red). The numbers of corresponding genes are shown at the top. (**B**) heatmap of 88 selected differentially expressed genes at translocation sites in T cells and NK cells. Gene names at translocation sites in T cells are indicated to the left and in NK cells to the right of the heatmap. The complete heatmap is shown in Figure S4. The values of the color key correspond to TPM. (**C**) the search of the genes at translocation sites was performed using the Enrichr Submissions TF-Gene Coocurrence Enrichr Submissions TF-Gene Coocurrence resource. Input genes are the genes at translocation sites. Enriched terms indicate the transcription factors that jointly regulate genes.

Previously, it was observed that active rDNA units possessing Pleiades often shape inter-chromosomal contacts with the genomic regions also acquiring hot spots of DSBs [5,6]. It was suggested that closely located chromosomal regions that both possess DSBs might give rise to incorrect end-joining and translocation. To determine whether transcribed genes, which may have more DSBs, are more often involved in rDNA-mediated translocation, we compared the expression of all genes and the expression of translocated genes. The violin plots shown in Figure 4A indicate that in both the normal T cells and in normal NK cells, the translocated genes are more actively transcribed than the bulk genes.

Among the bulk genes, there is a large proportion of silenced genes, while at translocation sites, there are fewer silenced genes, and the sites are enriched with moderately expressed genes. Table S1 shows the expression levels of the genes at translocation sites in both normal T cells and NK cells. We used normal NK cells because translocations in these cells might result in the appearance of an initial cancer cell that gives rise to a lymphoma.

The genes at translocation sites in both T cells and normal NK cells show different expression levels between the cell types. Figure 4B shows the heatmap of 88 selected differentially expressed genes located at translocation sites in both cell types. We found that often the genes that are more active in NK cells are less active in T cells and vice versa. For example, the *OASL* gene, which encodes dsRNA binding factor, is more actively transcribed in NK cells than in T cells. In contrast, *ZEB1-AS1* and *MAP3K14*, which are involved in histone modifications and in NF-kappaB signaling, respectively, are more active in T cells.

The complete heatmap (Figure S4) shows that are also many genes that have the same expression levels in both cell types. It follows that translocations in T cells and NK cells occur in genes with a wide range of expression levels. We determined whether there is a correlation between expression levels and the number of translocations using the data shown in Table S1. However, there was no correlation between the expression level and the translocation frequency. Therefore, we conclude that what is important for these translocations is some level of transcription. Probably, the presence of an open chromatin structure is required for rDNA-mediated translocation, which is supported by the data on the depletion of silenced genes at translocation sites in both T and NK cells (Figure 4A). It also follows that rDNA-contacting genes are regulated by different factors and have different expression levels.

Genes at translocations sites in T cells and NK cells are jointly regulated by different transcription factors. Figure 4C shows that up to ten transcription factors are involved in the regulation of these genes and seven transcription factors are common for different sets of genes in T cells and NK cells. The data suggest that the rDNA-contacting genes in T cells and NK cells are regulated by many transcription factors that are required for tuning of their expression levels in differentiated cells.

### 3.5. Epigenetic Features at Translocation Sites in T Cells and NK Cells

**Figure 5:**
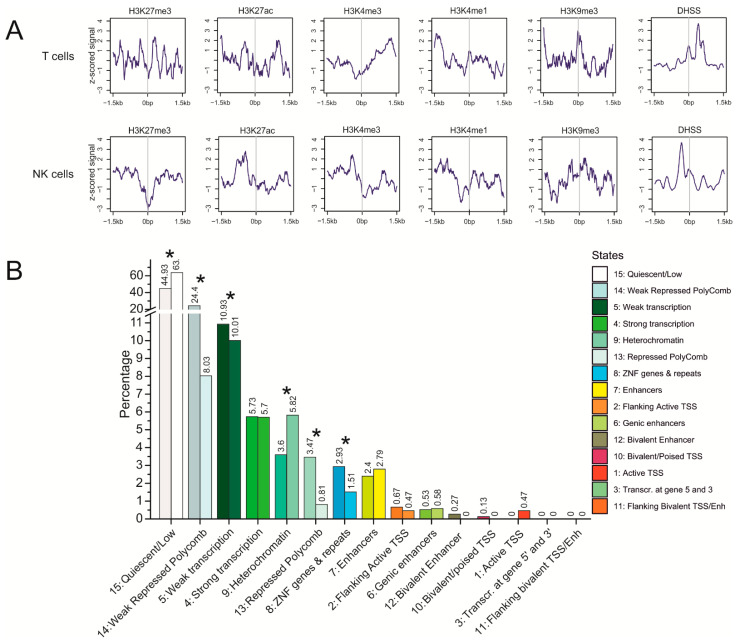
Properties of translocation targets in T cells and NK cells. (**A**) profiles of histone marks and DNase I hypersensitive sites (DHSS) around translocation sites. The z-scored signals ± 1.5 kb around translocation targets are indicated. (**B**) the ratios of chromatin states (15-state model) at translocation sites in T cells (right, brighter bars) and in NK cells (left, darker bars). The labels present a state number and the percentage of the corresponding state. * *p*-value < 0.0003.

To determine whether the translocation sites possess open chromatin structures, we analyzed the epigenetic states at translocations sites. The frequencies of translocations in T cells and NK-cell lymphomas were similar (Table 1) and thus, we assume that the translocations observed in NK-cell lymphomas correspond to the events in the normal NK cells. It follows that different sets of moderately expressed genes are targets of rDNA-mediated translocations in the normal T cells and NK cells (Table S1, Figure 4A). The detection of translocations indicates that these sets of genes have one property in common, that is, they shape the inter-chromosomal contacts within the active nucleoli. However, what are the local chromosomal factors that contribute to the translocations in different genomic regions in these cognate cell types? Do they share the same epigenetic features? Figure 5A shows the profiles of some histone modifications and the accessibility of chromatin ±1.5 kb around translocation sites in both cell types.

The repressive H3K27me3 mark associated with Polycomb-mediated gene silencing is depleted at the zero point of translocation sites in both cell types. However, in T cells, several peaks of this mark are located closely around, suggesting that Polycomb silencing around translocation sites is characteristic for T cells. The active histone marks (H3K27ac, H3K4me3, and H3K4me1) are characteristic of translocation sites in both cell types, although there are some differences in the distribution of these marks around the zero point of translocation sites. The active H3K27ac mark is characteristic of the hot spots of both rDNA-contacting sites and rDNA-mediated translocations (Figure 3). The heterochromatin-associated histone mark H3K9me3 is present in both cell types but is more prominent in T cells (Figure 5A). In both cell types, there are prominent DHSS peaks, indicating the presence of open chromatin areas close to the translocation sites, suggesting that access to the chromatin for rDNA-mediated translocation.

Figure 5B shows a comparison of the epigenetic states at the translocation sites in T cells and NK cells. In T cells, these sites are about four-fold enriched by the weakly repressed Polycomb state and about two-fold by the repressed Polycomb state, and by ZNF genes and repeats, compared with NK cells. At the same time, NK cells are enriched with heterochromatin. The characteristic differences in the chromatin states of translocation sites are statistically significant (*p* < 0.003). These differences in chromatin states between the two cell types suggest that rDNA contacts during differentiation occur within different genomic regions in different cell lines, which is why the patterns of translocated genes are different in the T cells and NK-cell lymphomas (Figure 2A).

## 4. Discussion

Translocations occur between closely located broken chromosomes. The critical distance between chromosomal regions essential for translocation is not known, but the fact that inter-chromosomal rDNA contacts, which were mapped at a resolution of ±2.5 kb [5], are involved in translocations suggests that this space is enough for a translocation event. Many different factors are also important for translocations: the frequencies of simultaneous DNA breakage in the contacting chromosomal regions, the accessibility of DNA sequences at both sites, and the dynamics of chromosomal contacts. We observed a good correlation between translocation frequencies for all nine hot spots of DSBs in rDNA units (R1–R9) in the normal and cancer cells (Figure 1C, Table 1), although the frequencies between different hot spots vary over a wide range. The data support the view that local chromosomal states are important for translocations. The dynamics of contacts are compartment-dependent and should be different for A and B compartments that correspond to active chromatin and silent chromatin, respectively [27]. Compartmentalization states that are formed by phase-separation mechanisms shaped by the attraction between chromatin domains of a similar state have differences in their chromatin interaction stabilities, as measured by the liquid chromatin Hi-C [28]. Pleiades are detected only in the active rDNA units, as far as the major γ-H2AX foci and UBF-1 binding sites are co-localized in interphase nuclei [6]. It follows that rDNA-mediated translocations should be enriched in open chromatin regions where active condensates are formed. The fact that *DUX4* genes, which are silenced and form frequent contacts with rDNA [29,30], are not involved in rDNA-mediated translocations in either T cells or NK-cell lymphomas support this supposition.

Recently, an unexpected diversity of NK cells with distinct transcriptomes was described, including a population of NK cells associated with the expression of rDNA [31]. The data are consistent with the regulatory role of nucleoli in the global regulation of gene expression. Previous results suggested that phase-separation mechanisms are involved in the inter-chromosomal interactions of rDNA units [30]. Growing evidence suggests the role of rDNA units in the global regulation of genes controlling development and differentiation [5,7,32,33,34]. We suppose that nucleoli contacts will be detected not only at the conserved multiple rDNA-contacting sites detected in different cell lines (Figure 3) but also in the regions of detected translocations in T and NK cells. The differences in the translocation sites in these cell types may depend on the patterns of genes that shape the contacts with the nucleoli during differentiation.

The etiology of NK-cell lymphoma is unknown, although there is a strong association with the Epstein–Barr virus [35]. No specific chromosomal translocation has been identified in NK-cell lymphomas, and a multi-omics study revealed several subtypes of NK-cell lymphomas that differ in their transcriptional signatures [36]. Three-dimensional chromosomal structures are highly variable between individual cells [37], which suggests that heterogeneity of the chromosomal architecture in NK cells could result in differences between tumorigenic translocations and lead to several subtypes of NK-cell lymphomas with different transcriptional signatures.

Translocations inside the big intron in the *CTCF* gene in NK-cell lymphomas (Figure S1, B) might not change expression of the gene, as tested by RNA-Seq. Nevertheless, such damage of *CTCF* could disrupt its function. Recently, it was shown that CTCF insulators serve as putative cancer drivers in different tumors, including lymphomas [38].

About 100 somatic translocations per genome were detected in both T cells and NK-cell lymphomas (Table 1). The majority of the genes associated with translocations did not overlap between the cell types (Figure 2A). We conclude that in T cells, translocations were not deleterious, and a particular translocation probably only affected a subset of T cells from the donor. Of course, all translocations are subjected to selection for viability in the corresponding cells and/or in their daughter cells. As a result, only some of the translocations should be present in a cancer stem cell if the translocations, or even a single translocation, are tumorigenic. During cancer progression, rDNA-mediated translocations in daughter cells could also occur. These novel translocations may affect a specific set of sites depending on the inter-chromosomal contacts of the nucleoli in cancer cells. As result, the differences in translocation patterns between populations of T cells and NK-cancer cells should increase. We suppose that these events are responsible for the observed discrepancy between the rDNA-mediated translocations in these two cell types.

Analysis of genomic features of lung cancer patients revealed mutational signatures in B lymphocytes that are coupled with distant chromosomal rearrangements, some of which correspond to fusions involving genes with important functions [26]. These data are consistent with our observation of translocations in normal and cancer cells. T cells and NK cells differentiate from the common lymphoid progenitor. Our data on the distinct rDNA-mediated translocation sites, which mainly originate from active chromosomal regions in T cells and NK-cell lymphomas, suggest that, even initially, the normal NK cells and T cells should have distinct patterns of expressed genes.

We conclude that in normal cells, rDNA-mediated translocation occurs in somatic cells and reflects the inter-chromosomal contacts of nucleoli with genes that are involved in development and differentiation. We estimate that the frequencies of these translocations are about 100 per genome for the entire population of T or NK cells in an individual. If a translocation is deleterious, a particular cell could be eliminated or give rise to a cancer stem cell. In general, all chromosomal contacts that are connected either with chromosome packaging or with the regulation of gene expression, coupled with DNA breakage, could lead to translocations and cancer. 

## 5. Conclusions

This study aims to uncover the role of rDNA-mediated translocation in tumorigenesis. It was predicted that the DSB hot spots in rDNA repeats that shape frequent inter-chromosomal contact with genes controlling differentiation and development could lead to translocations that give rise to cancer transformation. Analysis of normal T cells and NK-cell lymphomas from the same individuals showed that there are rDNA-mediated translocations in both cell types. However, the translocations in normal cells and tumor cells affect different set of genes that control development. Our data indicate that oncogenic rDNA-mediated translocations lead to dysregulation of a specific set of genes controlling development. The data suggest the role of rDNA units in cancer genesis.

## Figures and Tables

**Table 1:** rDNA sequences (anchors) used for searching for rDNA-mediated translocations and the detected frequencies of the translocations in T cells and NK-cell lymphomas.

Anchors	Sequences, 5′–3′	Numbering in rDNA Unit (Accession # U13369.1)	Average Translocation Number in T Cells (102 Samples)	Average Translocation Number in NK Cells Lymphomas (102 Samples)
R1	GCAGTGCGGTGGCGCGATCTTGGCTCACCGCAACCTCTGCCTCCCGGTTTCAAGCGATTCTCCTGCATCG	19,641–19,710	575.4	430.2
R2	CCTATTTTCAGTAGAGACGGGGTTTCTCCACGTTGGCCACGCTGGTCTCGAACTCCTGACCTCAAATGAT	21,261–21,330	829.6	881.9
R3	CTACTCGGGAGGCTGGGGTGGAAGAATTGCTTGAACCTGGCAGGCGGAGGCTGCAGTGAC	21,801–21,860	2015.5	2384.0
R4	TTTCTGAGATGGAGTCTTGCTCTGTCCCCCAGGCTGGAGTGCAGTGGCGT	31,701–31,750	1452.8	1723.9
R5	TGTCGCCCAGGCTGGAGTGCGATGGTGTGATCTCGGCTCACTGCAACCGCCACCTCCCTG	32,721–32,780	1733.0	1980.4
R6	CACCGCAACCTCCACCTCCCGCGTTCAAGCGATTCTCCTGCCTCAGCCTCCTGAGTAGCT	36,601–36,660	1154.2	1308.8
R7	CACTGCAACGTCCGCCTCCCGGGTTCACGCCATTCTCCTGCCTCAGCCTCCCAAGTAGCT	37,001–37,060	1.1	1.0
R8	AATCGCTTGAACCTGGGAAGCGGAGGTTGCAGTGAGCCGAGATTGCGCCATCGCACTCCA	38,551–38,610	856.7	885.1
R9	AGAGGGCAATGGCGCGATCTCGGCTCACCGCACCCTCCGCCTCCCAGGTTCAAGCGATTC	39,601–39,660	651.5	674.6
Total translocations in 102 samples			9269.8	10,269.9
Number of translocations per genome			90.9	100.68

## Data Availability

Not applicable.

## Supplementary Materials

The following are available online at https://www.mdpi.com/article/10.3390/cancers13153889/s1, Supplementary Methods: 4C procedure, Figure S1: Translocation sites in normal T cells and in NK cells in the regions of the *FHIT* and *CTCF* genes, Figure S2: TADs in the region of the *FHIT* gene, Figure S3: Translocation hot spots inside *LINC00486*, and Figure S4: The complete heatmap of differentially expressed genes at translocation sites in T cells and NK cells, Table S1: Genes at translocation sites in T cells and NK-cell lymphomas, Table S2: Overlap between genes located at translocation sites in T cells and in NK-cell lymphomas, Table S3: Genes located at rDNA-mediated translocation sites in T cells are involved in different biological processes (g:Profiler), Table S4: Genes located at rDNA-mediated translocation sites in NK-cell lymphomas are involved in different biological processes (g:Profiler).
