# Supplementary material for: Chromosomal Translocations in NK-Cell Lymphomas Originate from Inter-Chromosomal Contacts of Active rDNA Clusters Possessing Hot Spots of DSBs
Source: Cancers (Basel). 2021 Aug 2;13(15):3889. doi: 10.3390/cancers13153889 (PMC8345467; doi:10.3390/cancers13153889)
Supplement: Supplementary file 1 [file cancers-13-03889-s001.zip › cancers-1309637-supplementary.pdf]

cancers

MDPI

This file includes

Supplementary Methods
Supplementary Tables S1-S40
Supplementary Figures S1-S4

1. Supplementary Methods

1. 4C procedure

DNA samples were prepared as described previously (Dekker et al., 2002; Osborne et al., 2004; http://www.protocol-online.org/cgi-bin/prot/view_cache.cgi?ID=3978). To a suspension containing about 10×10^6 cells in 40 mL of cell culture medium, formaldehyde solution was added to final concentration 1.5%. After mixing, incubation of cell suspension was performed for 10 min at room temperature with mixing. Quenching with 2.75 mL of 2 M glycine (final concentration 0.125 M) was performed. After incubation at room temperature for 5 min the suspension was cooled for 15 min in an ice bath and then cells were collected by centrifugation for 15 min at 3500 rpm at 2°C. The pellet of cells was resuspended at 0 °C in 1 mL of buffer containing 10 mM Tris-HCl buffer, pH 8, 10 mM NaCl, 0.2% NP-40, and freshly added protease inhibitors (0.1 mM PMSF and 1:500 protease inhibitor cocktail (Sigma)). After incubation for 15 min, cells were homogenized by passing through a syringe about 50 times into an Eppendorf. Then nuclei were spun down by centrifugation for 5 min at 5000 rpm in an Eppendorf centrifuge 5415 R at 2°C.

The nuclei pellet was resuspended in 756 μL of solution containing 40 mM Tris-HCl buffer, pH 7.4, 50 mM NaCl, 10 mM MgCl2 and 10 mM 2-mercaptoethanol. Then 20% SDS was added to final concentration 0.3% and incubation with shaking was performed for 1 h at 37 °C. To sequester SDS, 180 μL of 10% Triton X-100 was added and the solution was incubated for 1 h at 37 °C. Before digestion, 1 μL of BSA (5 mg/mL) was added with mixing. Then 50 μL of EcoRI (10 u/μL) was added and after mixing digestion was performed overnight at 37 °C.

To inactivate the restriction enzyme, 35 μL of 20% SDS was added (final concentration 0.7%) and the probe was heated to 65 °C for 30 min. Then the mixture was transferred into a 15 mL Nunc tube and consequently 375 μL of 20% Triton X-100 (to final concentration 1%), 750 μL of 10× ligase buffer, 7.5 μL of BSA (5 mg/mL), 80 μL of 100 mM ATP, and 5241 μL of milliQ water were added and the final 7.5 mL solution was well mixed. Then 10 μL of T4 DNA ligase (200 u/μL) was added and after mixing incubation was performed for 5 h at 16 °C and then for 30 min at room temperature. During ligation, DNA concentration was equal to 2 ng/μL.

For isolation of DNA, 50 μL of proteinase K (10 mg/mL) was added (final concentration 50 μg/mL) and, after mixing, incubation was performed at 55 °C overnight. For RNA digestion, 40 μL of RNase A (10 mg/mL) was added (final concentration 0.5 μg/mL) and, after mixing, incubation was performed for 30 min at 37 °C. After extensive extraction with phenol-chloroform extraction (three times with 7 mL each) DNA was precipitated by 2.5 vol. of ethanol after addition of 40 μL 10 mg/ glycogen and 175 μL 4 M NaCl. The final DNA pellet was washed twice with 70% ethanol and then dissolved in 0.1×TE.

About 15 μg of DNA was digested in 250 μL solution with 75 u of FaeI overnight at 37 °C. Then the enzyme was inactivated by heating at 65 °C for 30 min. DNA was isolated after phenol-chloroform extraction, precipitated by ethanol, and dissolved in 100 μL of 0.1×TE.

For circularization, 15 μg of DNA was incubated in 8 mL of T4 DNA ligase buffer containing 400 u of T4 DNA ligase for 5 h at 16 °C. DNA was isolated after phenol-chloroform extraction, precipitated by ethanol, and dissolved in 50 μL of 0.1×TE. For preparation of 4C-rDNA libraries about 30 ng of 4C DNA preparations were used for 35 cycles of PCR with primers 5′ TCTTTGAAAAAAATCCCAGAAGTGGT 3′ and 5′

AAGTCCAGAAATCAACTCGCCAGT 3′ for each biological replicate. Deep sequencing of libraries were performed using HiSeq1500 (Illumina) using up to 150-nt long reads.

Dekker, J., Rippe, K., Dekker, M., et al. (2002). Capturing chromosome conformation. Science 95, 1306–1311.

Osborne, C.S., Chakalova, L., Brown, K.E., et al. (2004). Active genes dynamically colocalize to shared sites of ongoing transcription. Nat. Genet. 36, 1065–1071.

2. Supplementary Tables

Table S1. Genes at translocation sites in T cells and NK-cell lymphomas. Coordinates at translocation sites are shown according to hg19. The number of translocations corresponds to the number of WGS reads (amplicons) containing translocations. Some genes have more than one translocation site. There are 431 unique genes corresponding to translocations in T cells that are shown in 451 lines in the excel file. There are 487 unique genes corresponding to translocations in NK-cell lymphomas that are shown in 514 lines in the excel file. The excel file is attached separately.

Table S2. Overlap between genes located at translocation sites in T cells and in NK-cell lymphomas. Related to Venn Diagram in Figure 2A.

Copyright: © 2021 by the authors. Licensee MDPI, Basel, Switzerland. This article is an open access article distributed under the terms and conditions of the Creative Commons Attribution (CC BY) license (http://creativecommons.org/licenses/by/4.0/).

Names	Total	Genes
Lymphoma - T cells	35	*EDIL3 DLG2 PLCG2 CTNNA1 DIAPH2 DSCAML1 CNKSR3 PTH2R FBLN2 NLGN1 PTPRG KDM2A CNTNAP2 RP11-120I21.2 LINC00486 DNAH8 SYPL1 UNC5D TBXAS1 MEGF8 PCDH15 WBSCR17 ANO3 THSD4 FCHSD2 LAMA1 USP28 AC010127.3 E2F3 CTA-363E6.2 EFHC2 SETD3 CTD-2143L24.1 KCNQ1 MCTP1*
Lymphoma	451	*RP11-708B6.2 RAB2A CPA6 AC087645.1 RYR3 RP3-399L15.2 PIGX CLNK RALYL KIFC3 CTD-3179P9.1 INSL6 PTPRK PARVG RP5-945F2.3 AC013480.2 RNF38 AC002550.5 LIPF RCBTB1 CTD-3018O17.3 UBR1 CACNA1D WWC2 SOS1 SORCS1 ARSF CAMKMT SLC8A1-AS1 MALT1 SREK1IP1 RP11-392O1.4 RP11-752G15.3 RBFOX3 MOV10 HSPA8P13 RP11-436K8.1 C4orf22 EXT1 PTPRS LINC00276 ENPP4 SCYL2 TMEM51 GDA IGF2BP2 SLC4A8 BPIFB1 RAD51B RP11-12J10.3 TBC1D3H NCKAP5 NUP210L GATA3 RP11-111A22.1 FRY CHD1 PTPRN2 RP11-166B2.8 RP11-422J15.1 SUGCT EPS8 KCNT1 AKAP7 PCDH11Y TMPRSS9 LINC00469 ABL2 COMMD1 AP001605.4 BASP1 AC108868.5 CTC-340A15.2 LCLAT1 ZNF277 AGBL4 LIFR-AS1 WDFY3 DPM1 CCDC153 IAPP UTP18 ATL2 OGDH ZNF430 AP1S2 ANKH CORO7-PAM16 GAGE1 DSCAM SCOC WIPF2 FTO RP11-179A16.1 HMOX2 BIRC5 RAPGEF6 ADORA2A-AS1 ITGBL1 DAB1 GUCY1A2 RP11-21L1.1 CTD-2234N14.2 PPFIA2 PRKAR2A VWA5A LIFR FAM19A2 GBE1 RPS15A B4GALNT3 PGAP1 FOLH1 TTC28 MAGI2 CHD2 CACHD1 KDM4C AP001604.3 RNF4 RP11-126O1.4 CACNA1A SYT13 CTD-2536I1.1 BORA MAGI1-IT1 AC018462.2 LDLRAD4 ULK4 VWA8 NXF2 CNTRL RASAL1 RPS11P6 AC016768.1 RBFOX1 ZNF211 RP11-1029J19.5 CAMTA1 LINGO2 MAP3K14 RP11-525K10.3 RP13-726E6.1 EML4 CCDC144B HAO1 ENPEP*

		TAF12 AC003043.1 TUSC3 ATP11C C1orf168 HSD17B4 PCGF6 TBL1XR1 GAGE12I C15orf57 FEZ1 APOLD1 RP5-857K21.4 LARS PPIL2 SLC30A10 AP4S1 ZIM2 GALNT5 RP11-1096G20.5 SPTBN1 CAMK2D EFHC1 ABAT PI4K2A RBM47 NREP-AS1 RP11-540D4.3 DNAH9 ZFYVE28 GRIK2 IQSEC1 NAV1 RP11-6N13.1 STMND1 LTBP1 KIAA1549 SPINT1 AC023115.2 KIF16B RP11-308N19.1 PATL2 CDH2 GAGE2B KDM6A OTX2-AS1 MTDH CLSTN2 SPTAN1 RP11-13N12.1 RCAN2 TENM2 RP11-1220K2.2 GSPT1 MERTK AEBP2 PPAP2B NOMO1 TBX19 AKNAD1 ALG1L12P HTR3E-AS1 YTHDF2 LRMP RP13-884E18.4 RPL31P35 DCUN1D5 KIF15 AQP4-AS1 SLC9A3R1 RP11-64D24.2 SPANXA2-OT1 PRAMEF6 ZNRD1-AS1 TMEM189-UBE2V1 MROH9 SOX2-OT UBE2Q2P6 AP000289.6 CAMK1D CNGA1 NUMB TREH RP13-726E6.2 ZNF410 ZNF606 FAM126B RBPMS IQSEC2 RUSC2 C7orf63 CIRH1A WEE2-AS1 CCDC180 TMEM108 POLA1 FXYD2 RP11-425I13.3 PCDH9 HECW2 USP13 LPPR1 RP11-445O3.2 LINC00894 FSTL5 ARHGAP42 METTL10 KCNQ1OT1 CTC-535M15.2 TXNRD2 AC023669.1 RP11-417J8.2 TCP11X1 C11orf63 ZFPM2 FAF1 NAV3 GAGE2E KIAA1217 RDX AC074289.1 AC007796.1 ANKS1B ARHGEF6 CDH23 ERMAP NTM RP11-718O11.1 RP5-1021I20.5 RP11-359D14.3 FRMPD4 SPOCK3 FNTA RP11-321C24.1 UNC79 PREX2 C20orf166 BRD2 KCTD7 ITGB6 PTPRM RP11-170M17.1 RP11-192P3.4 AP000282.2 PLEKHS1 CADPS CLRN1-AS1 AC006115.3 ZEB1-AS1 ZNF800 RABGEF1 FBXL20 CORO7 TMEM189 NREP NFKBIZ BAGE2 C12orf56 TRAK1 TRIM51BP RP11-558F24.4 PPP2R5C FOXP1 CUEDC1 C16orf95 SPATA6 PRR5-ARHGAP8 RP11-23J9.5 SPAG6 KALRN GAGE12G LRRN4 NFKBID NEGR1 TBC1D3C RP11-35G9.5 NOVA1-AS1 INPP5B ARL15 TMEM232 RP11-739N20.2 SPATA13 MARS IPO9-AS1 NOX4 CABLES1 TCEB3 TTPAL NIPAL1 AC012593.1 ESR1 GCNT2 USP43 SLCO1A2 WDR78 CTC-432M15.3 ARID4B ECSIT GMDS PPP2R5E RP11-23J9.4 SDK2 FAM155A KCNAB1 ANKRD32 PDE4D RP11-307N16.6 VDAC1 TDRD9 AC016723.4 ATE1 CLIC2 NXF5 TRHDE RCAN3 WDR13 RPL9P8 HTR3E AC092684.1 OR2L13 ELL PSG11 PIK3CD DNER FMNL2 VPS13A NRXN1 RANBP2 RP11-42I10.1 AGBL1 ROR1 KIAA1432 SNHG14 AC010090.1 WWOX RUVBL2 LPHN2 UBE2D2 RP11-152F13.8 RP11-593F5.2 SPACA7 USP39 GAGE2D MGMT MUM1 MAGI1 JPH3 KNOP1 KAZN RP11-449D8.5 RNF128 CTNNA3 AP3B2 VPS13D DPP10 RAB4B RP11-248D7.2 GCNT1 OASL SPIN1 IFRD1 MIR3148 CTCF SNTB2 KB-1991G8.1 FUT8 RAB4B-EGLN2 CLASP2 ATP8B1 AC023115.1 RP11-598P20.5 KCNS3 AC096649.3 RP11-78B10.2 RP13-608F4.8 FMN2 TWSG1 RP11-32K4.1 PPM1D DPYSL3 SCN9A RGPD6 C1orf146 STYXL1 PRICKLE2 RP5-1021I20.4 RP11-450I1.4 NRROS PDLIM5 TRIM51EP RP11-607P23.1 RP11-35G9.3 C14orf164 KLHL32 RYBP CR1 ALPK2 RP11-24J23.2 ARHGAP8 TMTC2 C10orf11 NMRAL1 SPEF2 AC010880.1
T cells	384	RIT2 CDH3 HORMAD2 GPR155 GPHN GPR98 DOCK1 ATRX MYBL1 CRADD CREB3L2 RYK FRG2 CUBN CDC27 LINC01060 SEPT14 DCHS2 AMPH PCDHGA2 CTNND2 ST3GAL2 DNAH14 MYO9B RAP1A FAM126A FOXP2 KCNT2 SLC2A5 LGR6 BDH1 CLGN ERG RP11-707M1.1 RP11-142A5.1 PCDP1 RP11-452H21.1 RP11-734I18.1 RP11-17M15.2 PPP1R12B MIR181A1HG WDR70 RP11-707P17.2 SYNPO2 EZH1 TNS3 RORA EPDR1 MYH15 DENND2C SH3RF3 CTD-2540L5.5 CTC-268N12.3 UVRAG DKK2 LINC01141 RP11-73O6.3 TG ZNF717 MYO9A RP11-244B22.7 ACSL3 SLCO2B1 PLCB1 CPEB2 C3P1 OTX2 RP11-713M6.2 RP11-305F18.1 C1orf50 DMXL2 PPP3CA SERPINI1 RP11-886D15.1 RP11-71H9.1 TRIM13 RP11-65J21.4 CEACAM8 RP11-689K5.3 RASGEF1B CHODL-AS1 SNTG1 EXOSC7 PIH1D2 ANKRD20A3 GALK2 AKAP6 ITIH6 EXOC6B RP11-145E5.5 ABCB9 RP11-444A22.1 ISPD-AS1 PNPLA5 SLC22A15 C8orf44-SGK3 CTD-2058B24.2 LRRC58 LRP8 MEIS2 GNAQ PCDH7 IGSF1 OPCML RP11-218E20.2 GRM5 KIAA1324 PIBF1 ATG4C RP11-220C2.1 RP11-536C5.2 HMG20A LMF1 TMEM245 ABCB10 FNDC3A MRPL48 CELF1 RP11-574M7.1 HNRNPA1P54 RP11-795A2.2 PAOX RP5-843L14.1 CPEB4 B4GALT1 ESCO1 C21orf62

	LDHB MTAP HELLS FAM135B LHX6 CDK17 RP11-655H13.2 GATAD2A GSAP PLAC1 ITIH2 IFFO2 ZNF609 PCDHGA1 C4orf47 MMP26 PPP1CB RANBP17 CTB-43E15.2 PALM2 KRAS CCNG2 KTN1-AS1 GCKR SIGLEC14 CLEC3B RP11-2E17.1 RP11-40F8.2 PRKRIP1 EVI5 KCNQ5 MEF2BNB-MEF2B PDZD2 GFI1B ZNF407 RP11-371F15.3 AC012370.3 RP11-2F20.1 AC007091.1 LIPE-AS1 ZFYVE26 RP11-424D14.1 AP000233.4 RP11-702L6.4 SLC12A2 ISPD PALM2-AKAP2 RP11-160H12.2 MSH2 TIAM1 FOCAD CNR1 CTD-2280E9.1 SPATA6L AC093818.1 SORBS2 GLYATL2 PTPLB SCN1A CTD-3131K8.2 TJP3 MSRA NAB1 AC074391.1 TRPM3 TAF3 ANKRD20A11P TACR3 UBQLN1 RP11-53I6.2 RP11-427M20.1 DSCR4 NKAIN2 GTF2F2 SLC27A1 RALGAPA2 CCDC113 PIEZO2 RNF157-AS1 DTNB GPR158 LIMA1 RP5-867C24.5 TMEM132C C21orf49 LUC7L RP11-758M4.1 ZBED5-AS1 FAM114A2 RP11-115J23.1 COL23A1 NSD1 SIGLEC5 CTD-2562J17.2 RP11-745C15.2 RASAL2 RSPH1 MIPOL1 AC087239.1 CA5B ZNF98 SRBD1 GPATCH8 KIAA0930 PRKCQ KIF4A RP11-894J14.5 CCDC146 RNF10 SNCAIP RP4-537K23.4 CTD-2540L5.6 MAGOH RHAG CPS1 DNM1P47 SVOPL SPAG9 SIRPG PLD3 AKAP2 AP000959.2 FGD3 ZBTB8OS STAG3L2 TTC39C GPR141 ADAM9 MAD1L1 CYP7B1 ELMSAN1 USP25 DENND4A IFNAR1 RP11-146D12.2 RP11-488I20.9 SUMF1 SLC44A1 VPS11 KSR2 SIK3 RGS17 UBA6-AS1 GNG4 CNTNAP4 EF-CAB5 ELF4 TOMM40 ITGA6 C1GALT1 ZNF644 RP5-1006K12.1 RP11-260M19.2 SULF2 NRG2 LARP4 RP11-195B21.3 DNAJC1 AC062028.1 RP11-384F7.1 AXDND1 TLK2 FAM193B TMEM192 RP11-707P17.1 FBXL18 ARHGAP24 SMEK1 RNF157 IDE APTX CCT6B UBAP2 GYG1 TMPRSS11GP RP11-119K6.6 TJP1 ESRRB TBCD CSE1L PDSS2 AATF SLIT2 PCDH11X MEF2B LARS2 NMT2 UTP14A ZNF804A WFDC1 CSMD1 RP11-145G20.1 DOCK7 ETV6 GRAMD4 MAST4 SGK3 LYRM7 ATP6V0E2-AS1 RP11-665G4.1 GABRR3 PRKAG2 SPDYA FRMD4A ZNF223 AUTS2 SGCG MYO1D TYRO3 WASF3 PTDSS2 AC009784.3 AC005537.2 MARVELD2 EPB41L5 ZFAND6 RP11-286B14.1 AC096669.2 WDPCP ZFP82 FRG2B GAB2 BRAF IWS1 RP11-217C7.1 KIF5C KTN1 PASK RP11-380D23.2 SLC16A1 MIB1 SLC9B2 ADCY10 RP11-290K4.2 CDK3 RP13-279N23.2 PCDHGA3 EPHB1 GPC1 CCL24 AC013463.2 LPHN3 TEN1-CDK3 LINC00478 RPP38 LIMK2 EPCAM PATL1 DNAH2 FNBP1 CTC-552D5.1 AC131097.3 SENP5 KIAA1109 RP11-370I10.6 DNAJC10 KLF3

Table S3. Genes located at rDNA-mediated translocation sites in T cells are involved in different biological processes (g:Profiler). Related to Figure 1B.

GO.ID	Description	padj	Genes
		**Biological Process**	
GO:0048468	cell development	0.002806635416047701	*EPHB1,OTX2,RHAG,C1GALT1,LHX6,RAP1A,PCDH15,DSCAML1,TJP1,SPAG9,WDPCP,ZNF804A,GPC1,SERPINI1,PPP3CA,AUTS2,B4GALT1,KRAS,FNDC3A,TYRO3,ITGA6,RYK,NLGN1,SLC9B2,CTNND2,ATRX,DOCK7,DOCK1,TBCD,LAMA1,RIT2,RSPH1,SLIT2,MARVELD2,CTNNA1,CNR1,CNTNAP2,UNC5D,DIAPH2,LRP8,PRKCQ,CELF1,OPCML,RNF10,RNF157,MSH2,EPB41L5,PLCB1,SULF2,SORBS2,KIF5C,LGR6,MYO9A,CREB3L2,BRAF,GAB2,WASF3,AKAP6,TJP3,MEGF8,TIAM1,PTPRG,EFHC2*
GO:0048666	neuron development	0.010398150231535501	*EPHB1,OTX2,LHX6,RAP1A,PCDH15,DSCAML1,SPAG9,WDPCP,ZNF804A,GPC1,SERPINI1,PPP3CA,AUTS2,ITGA6,RYK,NLGN1,CTNND2,DOCK7,TBCD,LAMA1,RIT2,SLIT2,CTNNA1,CNR1,CNTNAP2,UNC5D,LRP8,PRKCQ,OPCML,RNF157,KIF5C,LGR*

			6,MYO9A,CREB3L2,BRAF,GAB2,MEGF8,TIAM1,PTPRG,EFHC2
GO:0031175	neuron projection development	0.02018738249924198	*EPHB1,OTX2,RAP1A,PCDH15,DSCAML1,SPAG9,ZNF804A,GPC1,SERPINI1,PPP3CA,AUTS2,ITGA6,RYK,NLGN1,CTNND2,DOCK7,LAMA1,RIT2,SLIT2,CTNNA1,CNR1,CNTNAP2,UNC5D,LRP8,PRKCQ,RNF157,KIF5C,LGR6,MYO9A,CREB3L2,BRAF,GAB2,MEGF8,TIAM1,PTPRG,EFHC2*
GO:0007155	cell adhesion	0.026271958150214405	*EPHB1,CNTNAP4,EPCAM,PCDH11X,PCDH15,DSCAML1,TJP1,WDPCP,SERPINI1,PCDH7,PPP3CA,DCHS2,PCDHGA1,PCDHGA2,PCDHGA3,EPDR1,B4GALT1,FNDC3A,TYRO3,CDH3,ITGA6,NLGN1,CTNND2,PDZD2,EDIL3,DOCK1,TBCD,LAMA1,CEACAM8,PPP1CB,FBLN2,CTNNA1,MAD1L1,CNTNAP2,UNC5D,PRKCQ,DLG2,OPCML,SIGLEC14,SIGLEC5,EPB41L5,SIRPG,ADAM9,BRAF,TJP3,TIAM1*
GO:0022610	biological adhesion	0.02961025647938043	*EPHB1,CNTNAP4,EPCAM,PCDH11X,PCDH15,DSCAML1,TJP1,WDPCP,SERPINI1,PCDH7,PPP3CA,DCHS2,PCDHGA1,PCDHGA2,PCDHGA3,EPDR1,B4GALT1,FNDC3A,TYRO3,CDH3,ITGA6,NLGN1,CTNND2,PDZD2,EDIL3,DOCK1,TBCD,LAMA1,CEACAM8,PPP1CB,FBLN2,CTNNA1,MAD1L1,CNTNAP2,UNC5D,PRKCQ,DLG2,OPCML,SIGLEC14,SIGLEC5,EPB41L5,SIRPG,ADAM9,BRAF,TJP3,TIAM1*
GO:0000902	cell morphogenesis	0.036807818816013985	*EPHB1,OTX2,PCDH15,DSCAML1,SPAG9,WDPCP,GPC1,PPP3CA,AUTS2,TYRO3,RYK,NLGN1,CTNND2,DOCK7,DOCK1,TBCD,LAMA1,SLIT2,CCL24,CNTNAP2,UNC5D,FGD3,LRP8,PRKCQ,LARP4,RNF157,EPB41L5,PALM2,KIF5C,LGR6,MYO9A,BRAF,GAB2,WASF3,MEGF8,TIAM1*
GO:0030054	cell junction	0.000029023488765478936	*CPEB4,EPHB1,SNCAIP,LIMA1,CNTNAP4,EPCAM,CPEB2,GNAQ,RAP1A,PCDH15,DSCAML1,TJP1,MIB1,ZNF804A,GPC1,PPP3CA,B4GALT1,KRAS,DMXL2,CDH3,ITGA6,NLGN1,ARHGAP24,SLC9B2,CTNND2,PDZD2,TNS3,DOCK7,FRMD4A,VPS11,TBCD,LAMA1,RIT2,PPP1CB,SCN1A,GABRR3,MARVELD2,CTNNA1,CNR1,AMPH,CNTNAP2,LRP8,SLC16A1,DLG2,MEF2B,DTNB,EPB41L5,PLCB1,SYNPO2,KIAA1109,SORBS2,MCTP1,ADAM9,FOCAD,FCHSD2,GRM5,GPHN,MYO9A,WASF3,AKAP6,TJP3,TIAM1,RGS17,SYPL1*
GO:0098590	plasma membrane region	0.0062905682244256715	*SNCAIP,SLC2A5,KCNQ1,LIMA1,CNTNAP4,EPCAM,FNBP1,ADCY10,TJP1,WDPCP,B4GALT1,ITGA6,NLGN1,SLC9B2,CUBN,IDE,GABRR3,MARVELD2,CTNNA1,CNR1,CNKSR3,CNTNAP2,LRP8,SLC16A1,DLG2,MYO1D,EPB41L5,SORBS2,ADAM9,FCHSD2,TACR3,GRM5,GPHN,SLCO2B1,AKAP6,SLC27A1,TIAM1,SLC12A2,SNTG1*

GO:0016323	basolateral plasma membrane	0.009528812238457019	*KCNQ1,EP-CAM,TJP1,B4GALT1,ITGA6,SLC9B2,IDE,MAR-VELD2,SLC16A1,DLG2,MYO1D,ADAM9,SLC27A1,SLC12A2*
GO:0070161	anchoring junction	0.013220132043484078	*LIMA1,EP-CAM,TJP1,PPP3CA,B4GALT1,KRAS,CDH3,ITGA6,ARHGAP24,CTNND2,PDZD2,TNS3,DOCK7,FRMD4A,TBCD,LAMA1,PPP1CB,SCN1A,MARVELD2,CTNNA1,CNT-NAP2,EPB41L5,SYNPO2,SORBS2,ADAM9,FO-CAD,AKAP6,TJP3,TIAM1*
GO:0005911	cell-cell junction	0.03398402330651312	*EP-CAM,TJP1,PPP3CA,B4GALT1,CDH3,ITGA6,ARHGAP24,CTNND2,PDZD2,FRMD4A,TBCD,LAMA1,SCN1A,MAR-VELD2,CTNNA1,CNT-NAP2,EPB41L5,AKAP6,TJP3,TIAM1*
GO:0042995	cell projection	0.04927092131241581	*CPEB4,EPHB1,DNAH14,LIMA1,OTX2,CNT-NAP4,CPEB2,SPATA6L,GNAQ,RAP1A,ADCY10,PCDH15,DSCAML1,TJP1,WDPCP,ZNF804A,PPP3CA,DNAH8,AUTS2,B4GALT1,KIF4A,ITGA6,NLGN1,ARHGAP24,SLC9B2,CTNND2,DOCK7,CUBN,RIT2,SCN1A,RSPH1,GABRR3,C4ORF47,CTNNA1,CNR1,CNT-NAP2,FGD3,LRP8,DLG2,MYO1D,EPB41L5,SORBS2,FAM126A,FCHSD2,TACR3,GRM5,KIF5C,GPHN,MYO9A,BRAF,WASF3,CCDC113,DNAH2,TIAM1,SLC12A2,RGS17,SNTG1,EFHC2*

Table S4. Genes located at rDNA-mediated translocation sites in NK-cell lymphomas are involved in different biological processes (g:Profiler). Related to Figure 1C.

GO.ID	Description	padj	GO.ID
		**Biological Process**	
GO:0048666	neuron development	1.4576027536988299e-8	*NEGR1,CTNNA1,PIK3CD,STYXL1,AGBL4,MOV10,CAMK1D,DSCAM,PDLIM5,STMND1,LAMA1,CDH2,PTPRS,CACNA1A,PTPRG,DPYSL3,ROR1,GATA3,CDH23,RASAL1,SLC9A3R1,SOS1,SPTBN1,TMEM108,TENM2,MAGI2,ABL2,PCDH15,TRAK1,OGDH,DAB1,PPFIA2,FRY,PTPRM,CLASP2,NLGN1,PTPRK,CNT-NAP2,UNC5D,EFHC2,SPTAN1,PREX2,EXT1,DSCAML1,FEZ1,NTM,SCYL2,NUMB,ATP8B1,MEGF8,NRXN1,HECW2,IQSEC1,ULK4,NREP*
GO:0030030	cell projection organi-zation	1.321205681790393e-7	*NEGR1,CTNNA1,PIK3CD,NAV3,STYXL1,EPS8,MOV10,CAMK1D,DNAH9,DSCAM,PDLIM5,RAPGEF6,STMND1,CNTRL,SPAG6,LAMA1,CDH2,PTPRS,CACNA1A,PTPRG,DPYSL3,ROR1,GATA3,CDH23,RASAL1,SLC9A3R1,SOS1,SPTBN1,TMEM108,TENM2,MAGI2,ABL2,PCDH15,TRAK1,DAB1,RDX,PPFIA2,FRY,PTPRM,CLASP2,NLGN1,PTPRK,CNT-NAP2,UNC5D,PARVG,ARHGEF6,EFHC2,SPATA13,SPTAN1,PREX2,EXT1,SPATA6,WDR78,DSCAML1,FEZ1,NUMB,ATP8B1,MEGF8,NRXN1,HECW2,IQSEC1,ULK4,SPEF2,NREP,DNAH8*

GO:0031175	neuron projection development	1.640061086819062e-7	*NEGR1,CTNNA1,PIK3CD,STYXL1,MOV10,CAMK1D,DSCAM,PDLIM5,STMND1,LAMA1,CDH2,PTPRS,CACNA1A,PTPRG,DPYSL3,ROR1,GATA3,CDH23,RASAL1,SLC9A3R1,SOS1,SPTBN1,TMEM108,MAGI2,ABL2,PCDH15,TRAK1,DAB1,PPFIA2,FRY,PTPRM,CLASP2,NLGN1,PTPRK,CNT-NAP2,UNC5D,EFHC2,SPTAN1,PREX2,EXT1,DSCAML1,FEZ1,NUMB,MEGF8,NRXN1,HECW2,IQSEC1,ULK4,NREP*
GO:0120036	plasma membrane bounded cell projection organization	9.861898816482843e-7	*NEGR1,CTNNA1,PIK3CD,NAV3,STYXL1,EPS8,MOV10,CAMK1D,DSCAM,PDLIM5,RAPGEF6,STMND1,CNTRL,LAMA1,CDH2,PTPRS,CACNA1A,PTPRG,DPYSL3,ROR1,GATA3,CDH23,RASAL1,SLC9A3R1,SOS1,SPTBN1,TMEM108,TENM2,MAGI2,ABL2,PCDH15,TRAK1,DAB1,RDX,PPFIA2,FRY,PTPRM,CLASP2,NLGN1,PTPRK,CNT-NAP2,UNC5D,ARHGEF6,EFHC2,SPATA13,SPTAN1,PREX2,EXT1,SPATA6,WDR78,DSCAML1,FEZ1,NUMB,ATP8B1,MEGF8,NRXN1,HECW2,IQSEC1,ULK4,SPEF2,NREP,DNAH8*
GO:0007399	nervous system development	0.000002019391019273909	*NEGR1,CTNNA1,TBX19,PIK3CD,NAV3,STYXL1,RBFOX3,AGBL4,MOV10,CAMK1D,SPINT1,DSCAM,PDLIM5,STMND1,RBFOX1,LAMA1,CDH2,PTPRS,CACNA1A,MERTK,PGAP1,PTPRG,KALRN,BASP1,DPYSL3,KDM4C,ROR1,GATA3,CDH23,RASAL1,SLC9A3R1,SOS1,SPTBN1,TMEM108,TENM2,MAGI2,ABL2,NAV1,PCDH15,TRAK1,OGDH,YTHDF2,DAB1,PPFIA2,FRY,PCDH9,PTPRM,CLASP2,CLSTN2,NLGN1,BRD2,PTPRK,CNT-NAP2,UNC5D,GDA,VPS13A,SDK2,TWSG1,ZNF430,NRROS,EFHC2,SPTAN1,PREX2,EXT1,DSCAML1,FEZ1,NTM,SCYL2,NUMB,ABAT,ATP8B1,MEGF8,NRXN1,HECW2,DNER,IQSEC1,ULK4,KCNAB1,IGF2BP2,SPEF2,NREP,EFHC1,LINGO2*
GO:0048699	generation of neurons	0.0000036661460672084216	*NEGR1,CTNNA1,PIK3CD,STYXL1,AGBL4,MOV10,CAMK1D,SPINT1,DSCAM,PDLIM5,STMND1,LAMA1,CDH2,PTPRS,CACNA1A,PTPRG,DPYSL3,KDM4C,ROR1,GATA3,CDH23,RASAL1,SLC9A3R1,SOS1,SPTBN1,TMEM108,TENM2,MAGI2,ABL2,NAV1,PCDH15,TRAK1,OGDH,YTHDF2,DAB1,PPFIA2,FRY,PTPRM,CLASP2,NLGN1,PTPRK,CNT-NAP2,UNC5D,SDK2,EFHC2,SPTAN1,PREX2,EXT1,DSCAML1,FEZ1,NTM,SCYL2,NUMB,ATP8B1,MEGF8,NRXN1,HECW2,DNER,IQSEC1,ULK4,NREP*
GO:0030182	neuron differentiation	0.00000421802939774706	*NEGR1,CTNNA1,PIK3CD,STYXL1,AGBL4,MOV10,CAMK1D,DSCAM,PDLIM5,STMND1,LAMA1,CDH2,PTPRS,CACNA1A,PTPRG,DPYSL3,KDM4C,ROR1,GATA3,CDH23,RASAL1,SLC9A3R1,SOS1,SPTBN1,TMEM108,TENM2,MAGI2,ABL2,PCDH15,TRAK1,OGDH,DAB1,PPFIA2,FRY,PTPRM,CLASP2,NLGN1,PTPRK,CNT-NAP2,UNC5D,SDK2,EFHC2,SPTAN1,PREX2,EXT1,D*

			SCAML1,FEZ1,NTM,SCYL2,NUMB,ATP8B1,MEGF8,NRXN1,HECW2,IQSEC1,ULK4,NREP
GO:0022008	neurogenesis	0.000009630331625955396	NEGR1,CTNNA1,PIK3CD,NAV3,STYXL1,AGBL4,MOV10,CAMK1D,SPINT1,DSCAM,PDLIM5,STMND1,LAMA1,CDH2,PTPRS,CACNA1A,PTPRG,DPYSL3,KDM4C,ROR1,GATA3,CDH23,RASAL1,SLC9A3R1,SOS1,SPTBN1,TMEM108,TENM2,MAGI2,ABL2,NAV1,PCDH15,TRAK1,OGDH,YTHDF2,DAB1,PPFIA2,FRY,PTPRM,CLASP2,NLGN1,PTPRK,CNT-NAP2,UNC5D,SDK2,NRROS,EFHC2,SPTAN1,PREX2,EXT1,DSCAML1,FEZ1,NTM,SCYL2,NUMB,ATP8B1,MEGF8,NRXN1,HECW2,DNER,IQSEC1,ULK4,NREP
GO:0048468	cell development	0.00007095407340787227	NEGR1,CTNNA1,PIK3CD,STYXL1,AGBL4,MOV10,CAMK1D,SPINT1,DSCAM,PDLIM5,STMND1,LAMA1,CDH2,PTPRS,CACNA1A,MERTK,PTPRG,DPYSL3,DI-APH2,ROR1,GATA3,CDH23,RASAL1,SLC9A3R1,SOS1,SPTBN1,TMEM108,HSD17B4,TENM2,ESR1,MAGI2,NUP210L,ABL2,FMN2,PCDH15,TRAK1,OGDH,YTHDF2,DAB1,ARID4B,RDX,PPFIA2,FRY,PTPRM,CLASP2,NLGN1,PTPRK,CNT-NAP2,UNC5D,PARVG,NRROS,PDE4D,EFHC2,SPTAN1,PREX2,EXT1,DSCAML1,FEZ1,NTM,SCYL2,NUMB,ATP8B1,ALPK2,MEGF8,NRXN1,HECW2,DNER,IQSEC1,ULK4,SPEF2,NREP
GO:0006928	movement of cell or subcellular component	0.0007225491786729031	CTNNA1,INPP5B,PIK3CD,NAV3,EPS8,AGBL4,CAMK1D,DNAH9,DSCAM,CTNNA3,LAMA1,LDLRAD4,CDH2,MERTK,CACNA1D,PTPRG,DPYSL3,GCNT1,GATA3,WIPF2,SLC9A3R1,SOS1,SPTBN1,TMEM108,MAGI2,ABL2,NAV1,FMN2,TRAK1,CAMK2D,OGDH,DAB1,RDX,FUT8,PTPRM,ITGB6,KIF16B,CLASP2,KIF15,ENPEP,PTPRK,UNC5D,VPS13A,KCNQ1,PDE4D,MCTP1,EFHC2,SPATA13,DLG2,SPTAN1,GCNT2,ITGBL1,AP3B2,KIFC3,EXT1,WDR78,NOX4,DSCAML1,FEZ1,NUMB,PLCG2,MEGF8,NRXN1,FMNL2,DNER,PPIL2,IQSEC1,ULK4,SPOCK3,SPEF2,DNAH8,EFHC1
GO:0000902	cell morphogenesis	0.0020097962737345276	PIK3CD,EPS8,MOV10,DSCAM,PDLIM5,LAMA1,CDH2,PTPRS,CACNA1A,MERTK,GATA3,RASAL1,SLC9A3R1,SOS1,SPTBN1,TMEM108,PCDH15,TRAK1,YTHDF2,DAB1,RDX,PPFIA2,FRY,PTPRM,ITGB6,CLASP2,NLGN1,CNT-NAP2,UNC5D,PARVG,SPTAN1,PREX2,EXT1,NOX4,DSCAML1,FEZ1,NUMB,MEGF8,NRXN1,FMNL2,HECW2
GO:0031344	regulation of cell projection organization	0.003538437498907764	NEGR1,NAV3,STYXL1,EPS8,MOV10,CAMK1D,DSCAM,PDLIM5,CDH2,PTPRS,CACNA1A,PTPRG,DPYSL3,ROR1,GATA3,TENM2,MAGI2,ABL2,TRAK1,DAB1,RDX,PPFIA2,NLGN1,EFHC2,FEZ1,ATP8B1,MEGF8,NRXN1,HECW2,ULK4
GO:0048812	neuron projection morphogenesis	0.004930178983282478	PIK3CD,MOV10,DSCAM,PDLIM5,LAMA1,CDH2,PTPRS,CACNA1A,GATA3,RASAL1,SOS1,SPTBN1,TMEM108,TRAK1,DAB1,PPFIA2,PTPRM,CLASP2,NLGN1,C

			NTNAP2,UNC5D,SPTAN1,PREX2,EXT1,DSCAML1,FEZ1,NUMB,MEGF8,NRXN1,HECW2
GO:0120035	regulation of plasma membrane bounded cell projection organization	0.00628295103971546 7	NEGR1,NAV3,STYXL1,EPS8,CAMK1D,DSCAM,PDLIM5,CDH2,PTPRS,CACNA1A,PTPRG,DPYSL3,ROR1,GATA3,TENM2,MAGI2,ABL2,TRAK1,DAB1,RDX,PPFIA2,NLGN1,EFHC2,FEZ1,ATP8B1,MEGF8,NRXN1,HECW2,ULK4
GO:0048667	cell morphogenesis involved in neuron differentiation	0.00643419897762428 6	PIK3CD,DSCAM,PDLIM5,LAMA1,CDH2,PTPRS,CACNA1A,GATA3,SLC9A3R1,SOS1,SPTBN1,PCDH15,TRAK1,DAB1,PPFIA2,PTPRM,CLASP2,NLGN1,UNC5D,SPTAN1,PREX2,EXT1,DSCAML1,FEZ1,NUMB,MEGF8,NRXN1,HECW2
GO:0007155	cell adhesion	0.00725829712902458 7	NEGR1,CTNNA1,DSCAM,FBLN2,PDLIM5,CTNNA3,LAMA1,CDH2,PTPRS,MERTK,GCNT1,CR1,GATA3,CDH23,MAGI1,TENM2,FAF1,ABL2,PCDH15,YTHDF2,DAB1,RDX,PPFIA2,PCDH9,PTPRM,MALT1,ITGB6,CLASP2,CLSTN2,NLGN1,PTPRK,CNTNAP2,UNC5D,SDK2,TWSG1,PARVG,DLG2,EDIL3,GCNT2,ITGBL1,KIFC3,EXT1,DSCAML1,FEZ1,NTM,ABAT,NFKBID,NRXN1,NFKBIZ,PCDH11Y
GO:0120039	plasma membrane bounded cell projection morphogenesis	0.00743088905963921 8	PIK3CD,MOV10,DSCAM,PDLIM5,LAMA1,CDH2,PTPRS,CACNA1A,GATA3,RASAL1,SOS1,SPTBN1,TMEM108,TRAK1,DAB1,PPFIA2,PTPRM,CLASP2,NLGN1,CNTNAP2,UNC5D,SPTAN1,PREX2,EXT1,DSCAML1,FEZ1,NUMB,MEGF8,NRXN1,HECW2
GO:0022610	biological adhesion	0.00828227638466711	NEGR1,CTNNA1,DSCAM,FBLN2,PDLIM5,CTNNA3,LAMA1,CDH2,PTPRS,MERTK,GCNT1,CR1,GATA3,CDH23,MAGI1,TENM2,FAF1,ABL2,PCDH15,YTHDF2,DAB1,RDX,PPFIA2,PCDH9,PTPRM,MALT1,ITGB6,CLASP2,CLSTN2,NLGN1,PTPRK,CNTNAP2,UNC5D,SDK2,TWSG1,PARVG,DLG2,EDIL3,GCNT2,ITGBL1,KIFC3,EXT1,DSCAML1,FEZ1,NTM,ABAT,NFKBID,NRXN1,NFKBIZ,PCDH11Y
GO:0048858	cell projection morphogenesis	0.00833522964234000 5	PIK3CD,MOV10,DSCAM,PDLIM5,LAMA1,CDH2,PTPRS,CACNA1A,GATA3,RASAL1,SOS1,SPTBN1,TMEM108,TRAK1,DAB1,PPFIA2,PTPRM,CLASP2,NLGN1,CNTNAP2,UNC5D,SPTAN1,PREX2,EXT1,DSCAML1,FEZ1,NUMB,MEGF8,NRXN1,HECW2
GO:0010975	regulation of neuron projection development	0.01170278205823662 4	NEGR1,STYXL1,CAMK1D,DSCAM,PDLIM5,CDH2,PTPRS,CACNA1A,PTPRG,DPYSL3,ROR1,GATA3,MAGI2,ABL2,TRAK1,DAB1,PPFIA2,NLGN1,EFHC2,FEZ1,MEGF8,HECW2,ULK4
GO:0098609	cell-cell adhesion	0.01184961056726677 9	NEGR1,CTNNA1,DSCAM,PDLIM5,CTNNA3,CDH2,PTPRS,GCNT1,CR1,GATA3,CDH23,MAGI1,TENM2,PCDH15,YTHDF2,DAB1,RDX,PCDH9,PTPRM,MALT1,CLSTN2,NLGN1,UNC5D,SDK2,TWSG1,DLG2,GCNT2,KIFC3,EXT1,DSCAML1,ABAT,NFKBID,NRXN1,NFKBIZ,PCDH11Y
GO:0043113	receptor clustering	0.01289469231760449 8	CDH2,CACNA1A,FNTA,MAGI2,NLGN1,DLG2,NRXN1,GRIK2

GO:0032990	cell part morphogenesis	0.014183882668813756	*PIK3CD,MOV10,DSCAM,PDLIM5,LAMA1,CDH2,PTPRS,CACNA1A,GATA3,RASAL1,SOS1,SPTBN1,TMEM108,TRAK1,DAB1,PPFIA2,PTPRM,CLASP2,NLGN1,CNTNAP2,UNC5D,SPTAN1,PREX2,EXT1,DSCAML1,FEZ1,NUMB,MEGF8,NRXN1,HECW2*
GO:0007275	multicellular organism development	0.01627227384198608	*NEGR1,CTNNA1,INPP5B,TBX19,PIK3CD,NAV3,STYXL1,RBFOX3,AGBL4,MOV10,CAMK1D,SPINT1,DSCAM,PDLIM5,STMND1,KAZN,RBFOX1,FTO,LAMA1,LDLRAD4,CDH2,PTPRS,CACNA1A,LCLAT1,MERTK,PGAP1,PTPRG,KALRN,BASP1,DPYSL3,KDM4C,GCNT1,DIAPH2,ROR1,CR1,GATA3,CDH23,RASAL1,SLC9A3R1,ELL,SOS1,SPTBN1,TMEM108,TBL1XR1,HSD17B4,TENM2,ESR1,MAGI2,AKNAD1,NUP210L,ABL2,NAV1,FMN2,KIAA1217,PCDH15,TREH,LTBP1,TRAK1,ANKH,OGDH,MTDH,ZFPM2,WDFY3,YTHDF2,DAB1,ARID4B,PPFIA2,FRY,PCDH9,FUT8,CHD2,PTPRM,MALT1,ITGB6,KIF16B,CLASP2,CLSTN2,NLGN1,ENPEP,BRD2,PTPRK,CNTNAP2,UNC5D,RNF38,GDA,VPS13A,KCNQ1,TDRD9,SDK2,TWSG1,ZNF430,NRROS,MGMT,EFHC2,FSTL5,SPTAN1,PREX2,WWOX,EDIL3,GCNT2,EXT1,SPATA6,WDR78,NOX4,DSCAML1,FEZ1,NTM,APOLD1,SCYL2,NUMB,ABAT,PLCG2,ATP8B1,ALPK2,NFKBID,MEGF8,NRXN1,SCN9A,HECW2,DNER,IQSEC1,ULK4,PRICKLE2,RYBP,NFKBIZ,KCNAB1,IGF2BP2,RBM47,SPEF2,NREP,EFHC1,LINGO2,NXF5,ATP11C*
GO:0000904	cell morphogenesis involved in differentiation	0.020734516172548123	*PIK3CD,DSCAM,PDLIM5,LAMA1,CDH2,PTPRS,CACNA1A,MERTK,GATA3,SLC9A3R1,SOS1,SPTBN1,PCDH15,TRAK1,YTHDF2,DAB1,PPFIA2,PTPRM,CLASP2,NLGN1,UNC5D,PARVG,SPTAN1,PREX2,EXT1,DSCAML1,FEZ1,NUMB,MEGF8,NRXN1,HECW2*
GO:0051179	localization	0.026618848969612804	*CTNNA1,AP1S2,INPP5B,CTCF,PIK3CD,NAV3,EPS8,CLNK,AGBL4,CACHD1,CAMK1D,PI4K2A,AP4S1,BIRC5,RAPGEF6,VDAC1,UBE2D2,E2F3,CNTRL,CTNNA3,SYT13,RYR3,RBFOX1,FTO,LAMA1,LDLRAD4,CDH2,CACNA1A,RUVBL2,MERTK,PGAP1,CACNA1D,PTPRG,KALRN,NIPAL1,CNGA1,DPYSL3,KCTD7,RABGEF1,FNTA,GCNT1,CR1,GATA3,CDH23,JPH3,WIPF2,SLC9A3R1,SOS1,SPTBN1,TMEM108,HSD17B4,ESR1,MAGI2,VPS13D,FAF1,ABL2,NAV1,FMN2,FCHSD2,KCNS3,LTBP1,DPP10,FAM126B,TRAK1,HTR3E,CAMK2D,ANKH,OGDH,DAB1,SLC30A10,ANO3,RDX,PPFIA2,ANKS1B,FUT8,PTPRM,MALT1,RANBP2,ITGB6,KIF16B,CLASP2,KIF15,NLGN1,ENPEP,ENPP4,PTPRK,CNKSR3,CNTNAP2,UNC5D,VPS13A,IQSEC2,RNF128,KCNQ1,LRMP,BORA,HAO1,NRROS,PDE4D,MCTP1,PTPRN2,EFHC2,SPATA13,DLG2,FAM155A,SLCO1A2,HMOX2,TBC1D3C,TBC1D3H,SPTAN1,AKAP7,SORCS1,GCNT2,IT*

			GBL1,AP3B2,CORO7,RPS15A,EXT1,NOX4,FXYD2,FEZ1,APOLD1,SLC4A8,SCYL2,NUMB,UNC79,ABAT,PLCG2,FBXL20,ATP8B1,RAB4B,RAB4B-EGLN2,MEGF8,NRXN1,COMMD1,FMNL2,SCN9A,HECW2,DNER,PPIL2,IQSEC1,ULK4,CADPS,KCNAB1,IGF2BP2,SPOCK3,SPEF2,DNAH8,EFHC1,GRIK2,TUSC3,RAB2A,KCNT1,NXF5,ATP11C,CLIC2
GO:0048856	anatomical structure development	0.027085999576717954	NEGR1,CTNNA1,INPP5B,TBX19,PIK3CD,NAV3,STYXL1,EPS8,RBFOX3,AGBL4,MOV10,CAMK1D,SPINT1,DSCAM,PDLIM5,VDAC1,STMND1,KAZN,RBFOX1,FTO,LAMA1,LDLRAD4,CDH2,PTPRS,CACNA1A,LCLAT1,MERTK,PGAP1,PTPRG,KALRN,BASP1,DPYSL3,KDM4C,GCNT1,DI-APH2,ROR1,CR1,GATA3,CDH23,RASAL1,SLC9A3R1,ELL,SOS1,SPTBN1,TMEM108,TBL1XR1,HSD17B4,TENM2,ESR1,MAGI2,AK-NAD1,NUP210L,ABL2,NAV1,FMN2,KIAA1217,PCDH15,TREH,LTBP1,TRAK1,ANKH,OGDH,MTDH,ZFPM2,WDFY3,YTHDF2,DAB1,ARID4B,RDX,PPFIA2,FRY,PCDH9,FUT8,CHD2,PTPRM,MALT1,ITGB6,KIF16B,CLASP2,CLSTN2,NLGN1,ENPEP,BRD2,PTPRK,CNT-NAP2,UNC5D,RNF38,GDA,VPS13A,KCNQ1,TDRD9,SDK2,TWSG1,ZNF430,PARVG,NRROS,PDE4D,MGMT,EFHC2,FSTL5,SPTAN1,PREX2,WWOX,EDIL3,GCNT2,RCAN3,EXT1,SPATA6,WDR78,NOX4,DSCAML1,FEZ1,NTM,APOLD1,SCYL2,NUMB,SETD3,ABAT,PLCG2,ATP8B1,ALPK2,NFK-BID,MEGF8,NRXN1,FMNL2,SCN9A,HECW2,DNER,IQSEC1,ULK4,PRICKLE2,RYBP,NFK-BIZ,KCNAB1,IGF2BP2,RBM47,SPEF2,NREP,EFHC1,LINGO2,NXF5,ATP11C
GO:0032409	regulation of transporter activity	0.03748681485466443	CACNA1D,KCTD7,JPH3,SLC9A3R1,CAMK2D,NLGN1,CNKSR3,KCNQ1,PDE4D,DLG2,AKAP7,FXYD2,PLCG2,NRXN1,HECW2,KCNAB1,CLIC2
GO:0048869	cellular developmental process	0.03765941629619539	NEGR1,CTNNA1,TBX19,PIK3CD,NAV3,STYXL1,AGBL4,MOV10,CAMK1D,SPINT1,DSCAM,PDLIM5,VDAC1,STMND1,KAZN,RBFOX1,FTO,LAMA1,LDLRAD4,CDH2,PTPRS,CACNA1A,MERTK,PTPRG,BASP1,DPYSL3,KDM4C,DI-APH2,ROR1,CR1,GATA3,CDH23,RASAL1,SLC9A3R1,SOS1,SPTBN1,TMEM108,TBL1XR1,USP13,HSD17B4,TENM2,ESR1,MAGI2,AK-NAD1,NUP210L,ABL2,NAV1,FMN2,PCDH15,TRAK1,OGDH,ZFPM2,YTHDF2,DAB1,SLC30A10,ARID4B,RDX,PPFIA2,FRY,PTPRM,MALT1,ITGB6,CLASP2,NLGN1,PTPRK,CNT-NAP2,UNC5D,TDRD9,SDK2,TWSG1,PARVG,NRROS,PDE4D,MGMT,EFHC2,PRAMEF6,FSTL5,IAPP,SPTAN1,PREX2,WWOX,GCNT2,EXT1,SPATA6,WDR78,NOX4,DSCAML1,FEZ1,NTM,APOLD1,SCYL2,NUMB,SE

			TD3,PLCG2,ATP8B1,ALPK2,NFK-BID,MEGF8,NRXN1,HECW2,DNER,IQSEC1,ULK4,NFKBIZ,KCNAB1,RBM47,SPEF2,NREP,ZNF277,ATP11C
GO:1904062	regulation of cation transmembrane transport	0.039979774288994914	*VDAC1,CACNA1D,JPH3,SLC9A3R1,DPP10,CAMK2D,NLGN1,CNKSR3,KCNQ1,PDE4D,DLG2,AKAP7,FXYD2,PLCG2,NRXN1,COMMD1,HECW2,KCNAB1,CLIC2*
GO:0032412	regulation of ion transmembrane transporter activity	0.0459027733909766	*CACNA1D,JPH3,SLC9A3R1,CAMK2D,NLGN1,CNKSR3,KCNQ1,PDE4D,DLG2,AKAP7,FXYD2,PLCG2,NRXN1,HECW2,KCNAB1,CLIC2*
GO:0097118	neuroligin clustering involved in postsynaptic membrane assembly	0.048249828474902716	*CDH2,MAGI2,NRXN1*
GO:0035609	C-terminal protein deglutamylation	0.048249828474902716	*AGBL4,FOLH1,AGBL1*
GO:0030154	cell differentiation	0.04967599471537364	*NEGR1,CTNNA1,TBX19,PIK3CD,NAV3,STYXL1,AGBL4,MOV10,CAMK1D,SPINT1,DSCAM,PDLIM5,VDAC1,STMND1,KAZN,RBFOX1,FTO,LAMA1,LDLRAD4,CDH2,PTPRS,CACNA1A,MERTK,PTPRG,BASP1,DPYSL3,KDM4C,DI-APH2,ROR1,CR1,GATA3,CDH23,RASAL1,SLC9A3R1,SOS1,SPTBN1,TMEM108,TBL1XR1,USP13,HSD17B4,TENM2,ESR1,MAGI2,AK-NAD1,NUP210L,ABL2,NAV1,FMN2,PCDH15,TRAK1,OGDH,ZFPM2,YTHDF2,DAB1,ARID4B,RDX,PPFIA2,FRY,PTPRM,MALT1,ITGB6,CLASP2,NLGN1,PTPRK,CNTNAP2,UNC5D,TDRD9,SDK2,TWSG1,PARVG,NRROS,PDE4D,MGMT,EFHC2,PRAMEF6,FSTL5,IAPP,SPTAN1,PREX2,WWOX,GCNT2,EXT1,SPATA6,WDR78,NOX4,DSCAML1,FEZ1,NTM,APOLD1,SCYL2,NUMB,SETD3,PLCG2,ATP8B1,ALPK2,NFK-BID,MEGF8,NRXN1,HECW2,DNER,IQSEC1,ULK4,NFKBIZ,KCNAB1,RBM47,SPEF2,NREP,ATP11C*

3. Supplementary Figures

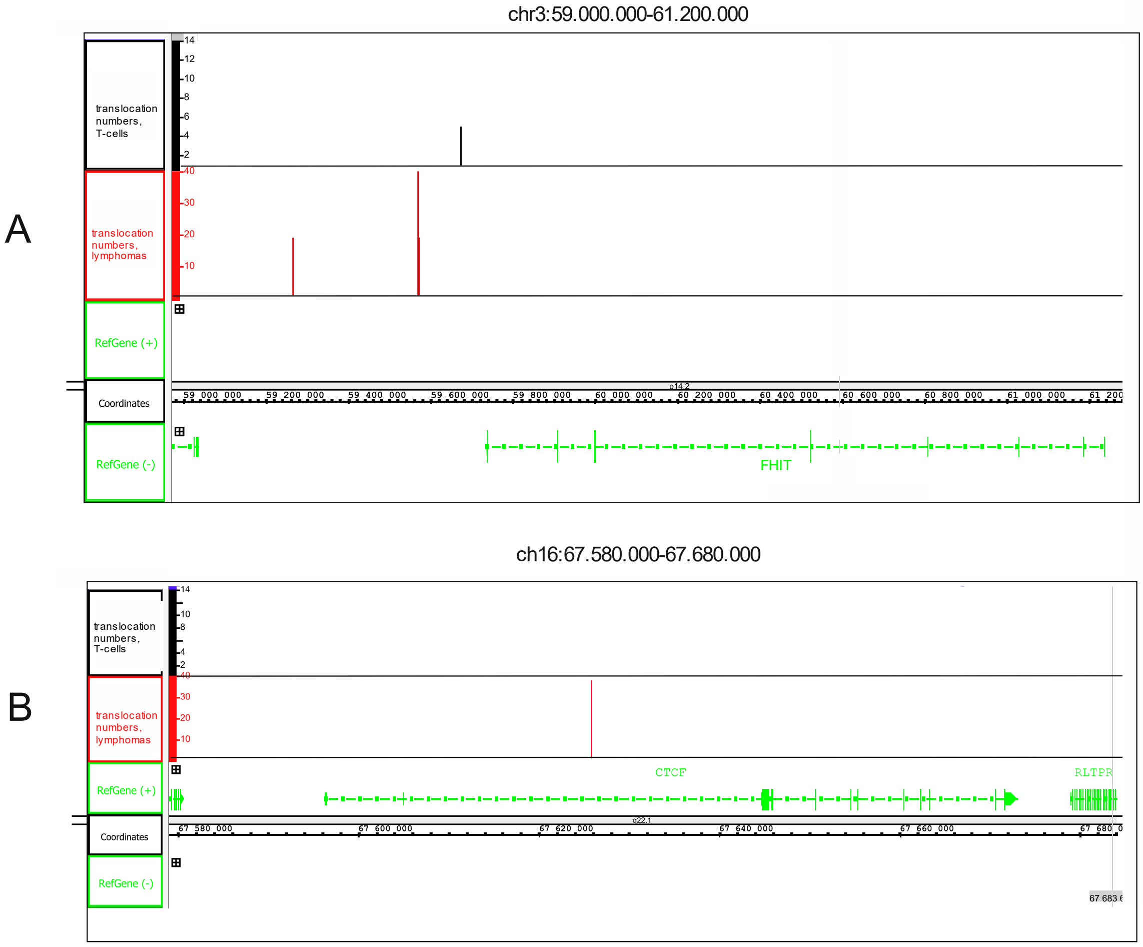

Figure S1. Translocation sites in normal T cells and in NK cells in the regions of the *FHIT* and *CTCF* genes. The translocation sites detected in 102 paired samples containing amplicons from T cells and NK-cell lymphomas from the same individual are shown. The distribution of genes is shown in green as in the UCSC Browser. There are two closely located (within about 2 kb) translocations sites at coordinate 59,57 Mb downstream of *FHIT*.

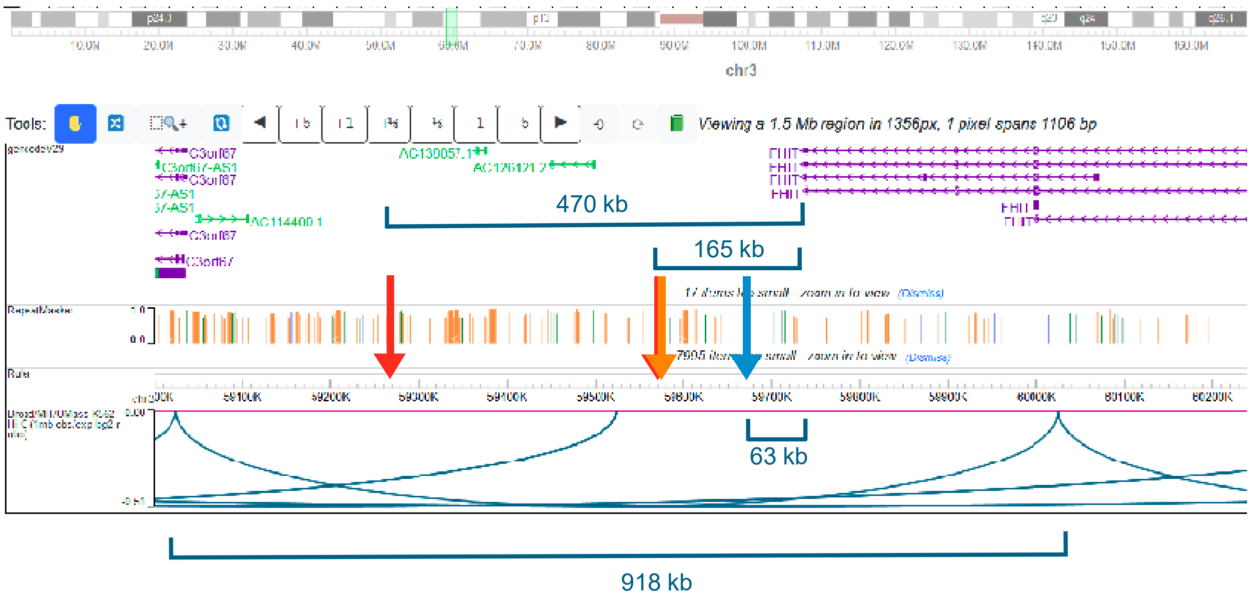

Figure S2. TADs in the region of the *FHIT* gene. The Wash EpiGenome Browser was used (http://epigenomegateway.wustl.edu/browser/). Two closely located (within about 2 kb) translocation sites at coordinate 59,57 Mb (hg19)

downstream of *FHIT* are shown by overlapping arrows in red and orange. The distances of translocations in T cells (blue arrow) and in NK-cell lymphomas (red or orange arrows) from the 3′ end of the gene are indicated by brackets. The TAD of 918 kb in length is shown at the bottom.

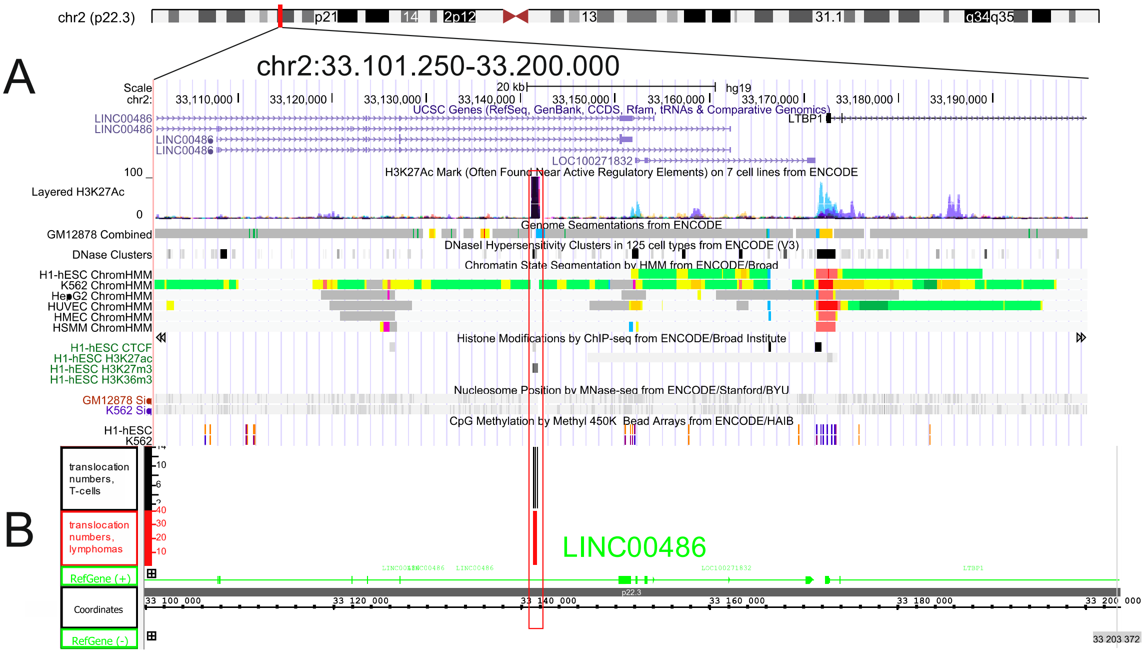

Figure S3. Translocation hot spots inside *LINC00486*. The translocation sites detected in 102 paired samples containing amplicons from T cells and NK-cell lymphomas from the same individual are shown. The distribution of genes is shown as in the UCSC Browser. The strong peak of the H3K27ac mark corresponds to the same sites of frequent translocations in both cell types.

Figure S4. The complete heatmap of differentially expressed genes at translocation sites in T cells and NK cells. The 451 genes from T cells and 514 genes from NK cells were analyzed. Expression values for the genes in TPM are shown in Table S1. The values in the color key correspond to TPM. The genes with translocations in T and NK cells are shown to the left and to the right of the heatmap, respectively. PDF file attached separately.
