# Supplementary material for: Chromosomal Translocations in NK-Cell Lymphomas Originate from Inter-Chromosomal Contacts of Active rDNA Clusters Possessing Hot Spots of DSBs
Source: Cancers (Basel). 2021 Aug 2;13(15):3889. doi: 10.3390/cancers13153889 (PMC8345467; doi:10.3390/cancers13153889)
Supplement: Supplementary file 1 [file cancers-13-03889-s001.zip › Figure S4.pdf]

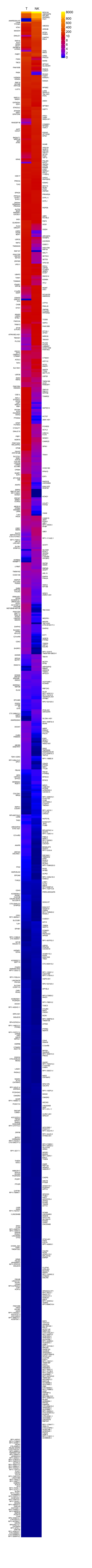
T
NK
6000
800
600
400
200
10
8
6
4
2
0
